# Functional loss of *rffG* and *rfbB,* encoding dTDP-glucose 4,6-dehydratase, alters colony morphology, cell shape, motility and virulence in *Salmonella* Typhimurium

**DOI:** 10.3389/fmicb.2025.1572117

**Published:** 2025-05-21

**Authors:** Subhashish Chakraborty, Pip Banerjee, Joel P. Joseph, Sanmoy Pathak, Taru Verma, Aagosh Kishor Karhale, Deepti Chandra, Mrinmoy Das, Pritam Saha, Dipankar Nandi

**Affiliations:** ^1^Department of Biochemistry, Indian Institute of Science, Bangalore, India; ^2^Department of Bioengineering, Indian Institute of Science, Bangalore, India

**Keywords:** lipopolysaccharide, enterobacterial common antigen, *Salmonella* Typhimurium, *rfbB*, *rffG*, dTDP-D-glucose 4, 6-dehydratase

## Abstract

Lipopolysaccharide (LPS) O-antigen and enterobacterial common antigen (ECA) play crucial roles in maintaining the outer membrane in Gram-negative bacteria. Mutations in the biosynthetic pathways of LPS and ECA may lead to accumulation of intermediates, resulting in morphological changes and activation of stress responses. However, the functional consequences of abrogation of both O-antigen and ECA synthesis in *Salmonella enterica* serovar Typhimurium (*S.* Typhimurium) are not well investigated. In this study, we generated single and double-deletion mutants of *rfbB* and *rffG*, encoding dTDP-glucose 4,6-dehydratase paralogs that are important in the synthesis of both O-antigen and ECA. Importantly, mutations in the dTDP-D-glucose 4,6-dehydratase encoding gene in humans are known to cause Catel-Manzke syndrome, a rare genetic disease. All four strains, i.e., wild type (WT), Δ*rfbB*, Δ*rffG* and Δ*rfbB*Δ*rffG*, grew well in rich Luria Bertani (LB) liquid media at 37°C; however, the functional loss of both *rfbB* and *rffG*, but not in single-deletion strains, resulted in round cell morphology and smaller colony size in LB agar plates. There was no significant differences in the growth of the four strains in minimal media at 37°C (nutritional deficiency), in LB at 42°C (high temperature), acidic pH or LB with 3–4% NaCl (high osmolarity; however the Δ*rfbB*Δ*rffG* strain was hypersensitive to bile and cell wall-targeting antibiotics). These results demonstrated that the Δ*rfbB*Δ*rffG* strain was sensitive to some stress conditions. Interestingly, the Δ*rfbB*Δ*rffG* strain displayed an altered LPS profile, autoaggregated rapidly compared to the WT and the single mutant strains and showed high N-phenylnaphthylamine (NPN) fluorescence indicating greater surface hydrophobicity. Furthermore, transcriptomic analysis identified flagellar and SPI-1 pathways to be highly downregulated in Δ*rfbB*Δ*rffG* which led to impaired swimming as well as swarming motility, lower adhesion and invasion of HeLa cells. Importantly, the Δ*rfbB*Δ*rffG* strain was less proficient in colonizing Peyer’s patches, spleen and liver, was unable to induce pro-inflammatory cytokines and was attenuated in both the oral and intraperitoneal models of *S.* Typhimurium infection in mice. Overall, this study highlights the importance of *rfbB* and *rffG* in maintaining cell wall and cell membrane integrity, colony and cellular morphology, motility and virulence in *S.* Typhimurium.

## Introduction

Salmonellosis is a global public health concern, especially with its rise as a food borne pathogen, increase in the number of antimicrobial resistant strains and high mortality by non-typhoidal strains due to co-infections in Africa (Kumar et al., 2025). The treatment of infections caused by *Salmonella* with the current generation of antibiotics continues to be a challenge, owing to the presence of the outer membrane. The outer membrane of Gram-negative bacteria is made up of lipopolysaccharide (LPS) and outer membrane proteins (OMPs). The presence of the LPS molecules provide a barrier which excludes toxic substances such as large hydrophobic antibiotics (Farrag et al., 2019). Another feature that is present on the outer membrane of species from the order Enterobacterales is the enterobacterial common antigen (ECA). ECA comprises repeating trisaccharide units composed of 4-acetamide-4,6 dideoxy D-galactose, N-acetyl-D-mannosaminuronic acid and N-acetyl-D-glucosamine (Gilbreath et al., 2012). The LPS, along with ECA, provides structural integrity to the outer membrane which is vital for the bacteria as their loss is associated with significant fitness cost (Spöring et al., 2018; Ilg et al., 2009).

*Salmonella enterica* serovar Typhimurium (*S.* Typhimurium) must overcome a wide array of host-mediated antimicrobial responses to establish successful infection in the host. LPS along with ECA form essential components of immunodominant molecules for these Gram-negative pathogens. Therefore, studying bacterial mutants deficient in LPS and ECA biosynthesis pathway offers a promising area of research. The enzyme, dTDP-glucose 4,6-dehydratase (RfbB) is involved in the dTDP-rhamnose biosynthesis pathway and is crucial in the formation of the LPS O-antigen, exopolysaccharides, capsular polysaccharides, and glycolipids (Supplementary Figure S1). This enzyme catalyzes the conversion of dTDP-*α*-D-glucose into dTDP-4-keto-6-deoxyglucose. The genes encoding the enzymes involved in the biosynthesis of O-specific polysaccharides are clustered in the *rfb* region (Supplementary Figure S2). However, another gene, *rffG*, is a paralog of *rfbB*, (Yao and Valvano, 1994) the product of which is another dTDP-glucose 4,6-dehydratase that catalyzes the same reaction as RfbB, but functions in the enterobacterial common antigen (ECA) pathway (Marolda and Valvano, 1995; Stevenson et al., 1994) (Supplementary Figure S1). A double deletion of these genes has been proposed to be a potential live vaccine candidate (Huang et al., 2016).

RfbB (also known as RmlB) functions as a homodimer and each monomer exhibits an *α*/*β* structure that is divided into two domains: the larger N-terminal domain that binds the nucleotide cofactor NAD^+^ while the C-terminal domain which is responsible for binding the sugar substrate, dTDP-D-glucose (Allard et al., 2002; Beis et al., 2003). The highly conserved active site, Tyr-X-X-X-Lys catalytic couple and the Gly-X-Gly-X-X-Gly motif at the N terminus characterize RfbB/RmlB as a member of the short-chain dehydrogenase/reductase (SDR) family. During glycan biosynthesis, TDP-dehydratase generates dTDP-rhamnose from dTDP-glucose. Undecaprenyl phosphate (UndP) and the UndP-linked oligosaccharides are important in LPS, capsular polysaccharides, peptidoglycan and additional pathways (Supplementary Figure S1). Consequently, disruptions in the biosynthetic pathways of LPS and ECA biosynthesis can lead accumulation of intermediates, resulting in morphological changes and activation of stress pathways (Castelli and Véscovi, 2011; Jorgenson and Young, 2016; Jorgenson et al., 2016; Castelli et al., 2008; Jorgenson and Bryant, 2021). Importantly, mutations in the dTDP-D-glucose 4,6-dehydratase encoding gene in humans are known to cause Catel-Manzke syndrome, a rare bone disease often characterized by abnormalities in the index finger (Ehmke et al., 2014).

Previously, our laboratory was involved in a collaborative study on the functional roles of UDP-glucose 4,6 dehydratase in *Candida albicans* (Sen et al., 2011). Studies conducted with the single-deletion mutants of either O-antigen or ECA have shown the importance of these two pathways on the physiology of *S.* Typhimurium (Rai Ashutosh and Mitchell Angela, 2020; Marshall and Gunn, 2015; Park et al., 2018; Huang et al., 2016). One study has shown that a strain harboring double deletion of *rffG* and *rfbB* has a potential to be a live vaccine candidate (Huang et al., 2016). However, there is paucity of information on the detailed characterization of the direct as well as indirect effects of the loss of both O-antigen and ECA in *S.* Typhimurium. With this objective in mind, we initiated this study and generated single and double-deletion mutants of *rffG* and *rfbB* to study the functional loss of dTDP-glucose 4,6-dehydratase. The broad objective of this study was to understand the consequences of the combinatorial loss of O-antigen and the ECA and its effects on the physiology and virulence of *S.* Typhimurium.

## Materials and methods

### Bacterial strains and growth conditions

The bacterial strains used in this study are listed in Supplementary Table S1. All bacterial cultures were grown in Luria-Bertani (LB) medium consisting of 10 g/L tryptone (HiMedia Laboratories, Mumbai, India), 10 g/L NaCl (Merck, Darmstadt, Germany), and 5 g/L yeast extract (HiMedia Laboratories) at 37°C. Strains containing pKD46 were cultured at 30°C with constant shaking at 160 rpm. LB containing antibiotics were used at the following concentrations: kanamycin, 50 μg/mL; chloramphenicol, 30 μg/mL; tetracycline, 10 μg/mL and/or ampicillin, 100 μg/mL.

### Generation of knockout strains and complementation

All single-deletion mutants used in this study were generated using the one-step gene disruption strategy (Datsenko and Wanner, 2000). *S.* Typhimurium 14028s (WT) was used as the parent strain for all experiments. For the construction of Δ*rffG*, primers listed in Supplementary Table S2 (Sigma, Bangalore, India) were designed to amplify the kanamycin cassette from the template, pKD4. The resulting PCR-amplified product was purified and electroporated into the WT strain harboring pKD46, which expresses the λ Red recombinase. A similar methodology was followed for generating the Δ*rfbB* strain, where the gene was replaced with the chloramphenicol cassette (pKD3 template). The double deletion strain, Δ*rfbB*Δ*rffG* was constructed by amplifying *rfbB* gene fragment and electroporating the amplicon into Δ*rffG* strain harboring pKD46. All the mutants were confirmed by PCR amplification using primers designed to anneal ~100 bp upstream and downstream of the gene (Supplementary Table S2).

### Cloning of genes for complementation

*Salmonella* Typhimurium 14028s genomic DNA was used as a template for the PCR amplification of genes *rffG* and *rfbB* with the specific primers (Supplementary Table S2) using the Phusion DNA polymerase. The genes, *rffG* and *rfbB* were cloned between the sites, NcoI and HindIII in the pACDH plasmid (Rao and Varshney, 2001). Positive clones were confirmed by Sanger sequencing (Aggrigenome, India) and transformed into appropriate strains for complementation.

### Stress assays

*Salmonella* Typhimurium WT, ∆*rfbB*, ∆*rffG* and ∆*rfbB*∆*rffG* strains were grown in 3 mL LB or LB with appropriate concentration of antibiotics overnight at 37°C with 160 rpm. The optical density at 600 nm (OD_600_) of the pre-inoculum was normalized to 2.0, and 50 μL of the culture (normalized pre-inoculum) was added to 5 mL of LB without or with different concentrations of either bile salts (Sigma Aldrich, USA), NaCl or different pH range. The cultures were incubated at 37°C under shaking conditions at 160 rpm for 6–8 h and OD_600_ were measured in an UV–Visible spectrophotometer (Shimadzu) (Ray et al., 2019).

### RNA isolation and cDNA synthesis

For total RNA preparation, bacterial cultures were grown for 3 h in LB, pelleted and stabilized with RNAprotect Bacteria Reagent (Qiagen) and stored at −80°C. Total RNA was extracted using TRIzol (Sigma, St. Louise, Missouri, USA). Subsequently, 2–5 μg DNase-treated RNA was reversed transcribed to cDNA using the RevertAid First Strand cDNA synthesis kit (Thermo Fisher Scientific, Walter, MA, USA) (Ray et al., 2019).

### Quantitative real-time PCR

The cDNA was diluted to 1:20 and analysed using iQ5 Real time PCR detection system (BioRad, Hercules, California, USA) with SYBR Green detection system. Each sample was kept in triplicates in a 96-well plate (BioRad, Hercules, California, USA) in total reaction mixture of 10 μL containing 2X SYBR iQ SYBR Green supermix and 10 μM primer mix. PCR conditions were as followed, 95°C for 5 min, followed by 39 cycles of 95°C for 30s, 57°C for 30s and 72°C for 30s. The amplification specificity and the primer dimer were calculated by the melt curve acquired after 81 cycles of heating PCR products from 55°C to 95°C for 20s, with 5°C increase per cycle. The untreated WT cells, at the respective time points were normalized using the reference control gene, *gmk*, and all other samples were calculated as fold change to this reference value, using the 2^-ΔΔCt^ method (Livak and Schmittgen, 2001).

To estimate qPCR primer efficiency, RNA was isolated from WT 14028s at the indicated time point using the TRIzol method. cDNA was synthesized as described previously and serially diluted at 1:10, 1:100, 1:1000, and 1:10,000. Real-time PCR was performed under the conditions mentioned above. The Ct values were plotted against the cDNA concentration, and the slope of the standard curve was calculated. Primer efficiency and amplification factor was determined using the formula: Efficiency = 10^(−1/slope)-1^ (Rogers-Broadway and Karteris, 2015). The primer efficiency was calculated to be in the range of 1.7–1.8, as described in Supplementary Figure S4.

### Atomic force microscopy

The bacterial cells were pelleted down after growth for indicated time by centrifugation at 6000 rpm and washed with double distilled water thrice. Around 5 μL was placed on top of a glass coverslip and the images were obtained by using NX-10 AFM (Park systems, South Korea) (Varghese et al., 2020; Verma et al., 2021). Cellular width was measured using the image processing software XEI (Park Systems, South Korea). For the Δ*rfbB*Δ*rffG* strain, which are spherical, although not perfect spheres, the cellular width was measured considering the smaller dimension to be the width of the cell.

### MIC determination

The MIC was determined by E-tests using Ezy-MIC strips (HiMedia, Mumbai, India) according to the manufacturer’s protocol. Briefly, the overnight grown cultures were normalized to 2.0 OD at 600 nm and spread-plated onto the Mueller Hinton agar plates by spread plate method. Pre-coated MIC strips with antibiotics were placed in the middle of the agar plates with the help of a sterile swab and the plates were left in an incubator maintained at 37°C for 18 h. The MIC was read at the point of the strip at which zone of clearance coincided with the strip (Bhaskarla et al., 2016).

### Analysis of the LPS profile

Bacterial cells were grown for 16 h, and the OD was normalized to 2.0 at 600 nm. Cells were centrifuged and the pellets were suspended in 150 mL of lysis buffer containing proteinase K (Thermo Scientific, Walter, MA, USA) followed by hot phenol extraction and a subsequent extraction of the aqueous phase with diethyl ether. LPS was separated on 12% (w/v) acrylamide gels using a Tricine-SDS buffer system and visualized by silver staining (Hitchcock and Brown, 1983).

### NPN assay

The outer membrane permeability was measured with the help of NPN assay as previously described (Spöring et al., 2018). Briefly, the cells were grown till OD of 0.5 at 600 nm and the cells were harvested by centrifugation. The pellet was washed with 5 mM HEPES (pH 7.2) and adjusted to an OD of 0.5 at 600 nm. NPN was added to each well at the final concentration of 10 μM. 200 μL was added to a flat bottom 96-well plate and fluorescence excitation and emission was measured at 350 nm and 420 nm, respectively.

### Auto-aggregation assay

The bacterial growth for every strain was normalized to OD 2.0 at 600 nm and the cultures were kept in a static, upright position for 30 min. At regular intervals of 10 min, 100 μL of the culture was collected from the top and the absorbance was measured at 600 nm. Absorbance at 600 nm versus time was plotted over a 30-min time interval (Raghunathan et al., 2011).

### Motility assays

Bacterial motility assays were performed as described previously (Ray et al., 2020; Thakur et al., 2020). Briefly, swimming motility assay was performed on a freshly prepared 0.3% agar whereas for swarming motility assays, 0.5% agar was used along with 0.5% glucose (HiMedia, Mumbai, India). For both types of motility assays, 2 μL of overnight culture normalized to 1 OD_600_ were inoculated in the center of the plates, and the plates were incubated at 37°C for 8 h and then imaged using the ImageQuant LAS4000 (GE Healthcare).

### HeLa cell and mice infections

The detailed protocol followed for HeLa cell infections are in the Supplementary methods. Six to eight-week old, male, C57BL/6 mice (*n* = 4–6 mice per group) were orally infected with ~1 × 10^8^ CFU/mouse or intra-peritoneally (i.p.) with ~1 × 10^3^ CFU/mouse of either the WT or the Δ*rfbB*Δ*rffG* strain (Majumdar et al., 2017). Organ bacterial burden was estimated 4 days after infection. The organs were harvested, weighed, and homogenized in 1 mL sterile PBS. Appropriate dilutions were plated on LB agar plates to enumerate CFU as log_10_(CFU/gram tissue weight).

### Measurement of cytokines in sera

The amounts of TNFα, IL6 and IFNγ in sera were quantified using sandwich ELISA (Yadav et al., 2018) using the appropriate kits (Thermo Fisher Scientific, USA). Sera were collected at day 4–5 post oral gavage or day 3 post i.p. injection. 3,3′,5,5’-Tetramethylbenzidine (TMB) was used as the chromogenic substrate, and absorbance was recorded at 450 nm using VersaMax Microplate Reader (Molecular Devices, USA).

### Other assays

The detailed protocols involved in multiple sequence alignment (MSA), RNA sequencing and data analysis, isolation of outer membrane proteins (OMP) and adhesion and invasion assays are described in detail in the Supplementary methods section.

### Statistical analysis

All graphs were plotted, and the statistical analyses performed using GraphPad Prism 8 (v 8.0.2) software (GraphPad, La Jolla, CA). For most experiments, statistical analyses were performed using either one-way or two-way ANOVA. Data are represented as mean ± SEM, where * *p* < 0.05, ∗∗ *p* < 0.01, ∗∗∗ *p* < 0.001 and ∗∗∗∗ *p* < 0.0001. For *in vivo* infection studies, statistical analyses of mice survival curves post infection were performed using the log-rank (Mantel-Cox) test. For estimation of cytokines, statistical analysis was performed using the Kruskal Wallis test, where * p < 0.05; ** p < 0.01 and *** *p* < 0.001.

## Results

### Multiple sequence alignment of RffG and RfbB protein homologs indicate presence of a conserved YXXXK motif

The genes under investigation, *rffG* and *rfbB* are paralogs, (Yao and Valvano, 1994) each encoding the enzyme dTDP-glucose 4,6-dehydratase (Supplementary Figure S1) and are organized as part of distinct operons (Supplementary Figure S2). A sequence analysis was performed to investigate the protein sequence conservation from lower to higher organisms and to also analyze whether the known functional motifs are conserved. To obtain homologs, the protein FASTA sequences of RfbB (361aa, locus id: STM14_2591) and RffG (355aa, locus id, STM14_4720) from *S.* Typhimurium 14028s were retrieved from NCBI. These sequences were then independently used as query sequences to perform protein BLAST against the target non-redundant protein sequence database. The hits obtained were further filtered. Multiple sequence alignment and representation of the aligned sequences were performed using CLUSTAL Omega and JalView, respectively. The crystal structure of dTDP-D-Glucose 4,6-dehydratase (RfbB or RmlB) from *S.* Typhimurium, shows two regions in the active site: the cavity created by the NAD^+^ binding region and the dTDP-D-glucose-binding regions (Allard et al., 2001). Mechanistic roles of the residues Thr133, Tyr167 and Lys171 in the catalytic motif of the enzyme have been studied through mutagenesis in *Escherichia coli* (Gerratana et al., 2001). We observed that eukaryotic homologs have a very low degree of sequence (25–40%) similarity. However, the Tyr (residue 167), part of the YXXXK motif is strictly conserved in this extended SDR family and is also the substrate binding site. Thr133 and Lys171 in RfbB are also highly, but not absolutely, conserved. Other highly conserved residues include the three glycine residues, with motif (Gly-X-Gly-X-X-Gly) located close to the N terminus (at positions 8, 10 and 13 in RfbB) are involved in NAD^+^ binding. Although the SDR signature motif (LftettayapSspYSASKASSdHLVrAWR) slightly varied in the RffG sequence, it did retain the active site Tyr and Lys residues (Supplementary Figure S3A). The other residues known to make contact with the nucleotide sugar are Thr133, Asp134, Glu135, Asn196, Arg231 and Asn266. Notably, amino acid variations were observed in *Shigella, Mycobacterium, Drosophila* and *Saccharomyces* species (Supplementary Figure S3). Overall, it was interesting to observe the presence of homologs of this enzyme from *E. coli* to humans.

### *S.* Typhimurium Δ*rfbB*Δ*rffG* strain shows higher optical density and a delayed lag phase compared to the WT and single-deletion mutants

To better understand the functional roles of *rffG* and *rfbB* in *S.* Typhimurium 14028s, single and double-deletion mutants were generated, and growth assays were performed. The WT and the single-deletion strains displayed similar growth kinetics; however, the Δ*rfbB*Δ*rffG* strain attained a slightly higher optical density (OD) than the other strains beginning from the mid-exponential phase till the stationary phase (Supplementary Figure S5A). Notably, introducing the WT copy of either *rffG* or *rfbB* restored the OD to WT levels (Supplementary Figure S5B). To further investigate whether the higher OD displayed by the Δ*rfbB*Δ*rffG* strain corresponded with the actual number of viable cells, appropriate dilutions were plated on agar plates at various time points. Cellular viability between the WT and the single-deletion mutants were not significantly different; however, the Δ*rfbB*Δ*rffG* strain at 3 h showed lesser CFU when compared to other strains (Supplementary Figure S5C). Apart from demonstrating a prolonged lag phase in the liquid medium wrt CFU, there were no other observable growth differences in the Δ*rfbB*Δ*rffG* strain compared to the WT and the single-deletion mutants.

### *S.* Typhimurium Δ*rfbB*Δ*rffG* forms smaller colonies and a distinct colony morphology on LB agar plates

**Figure 1:**
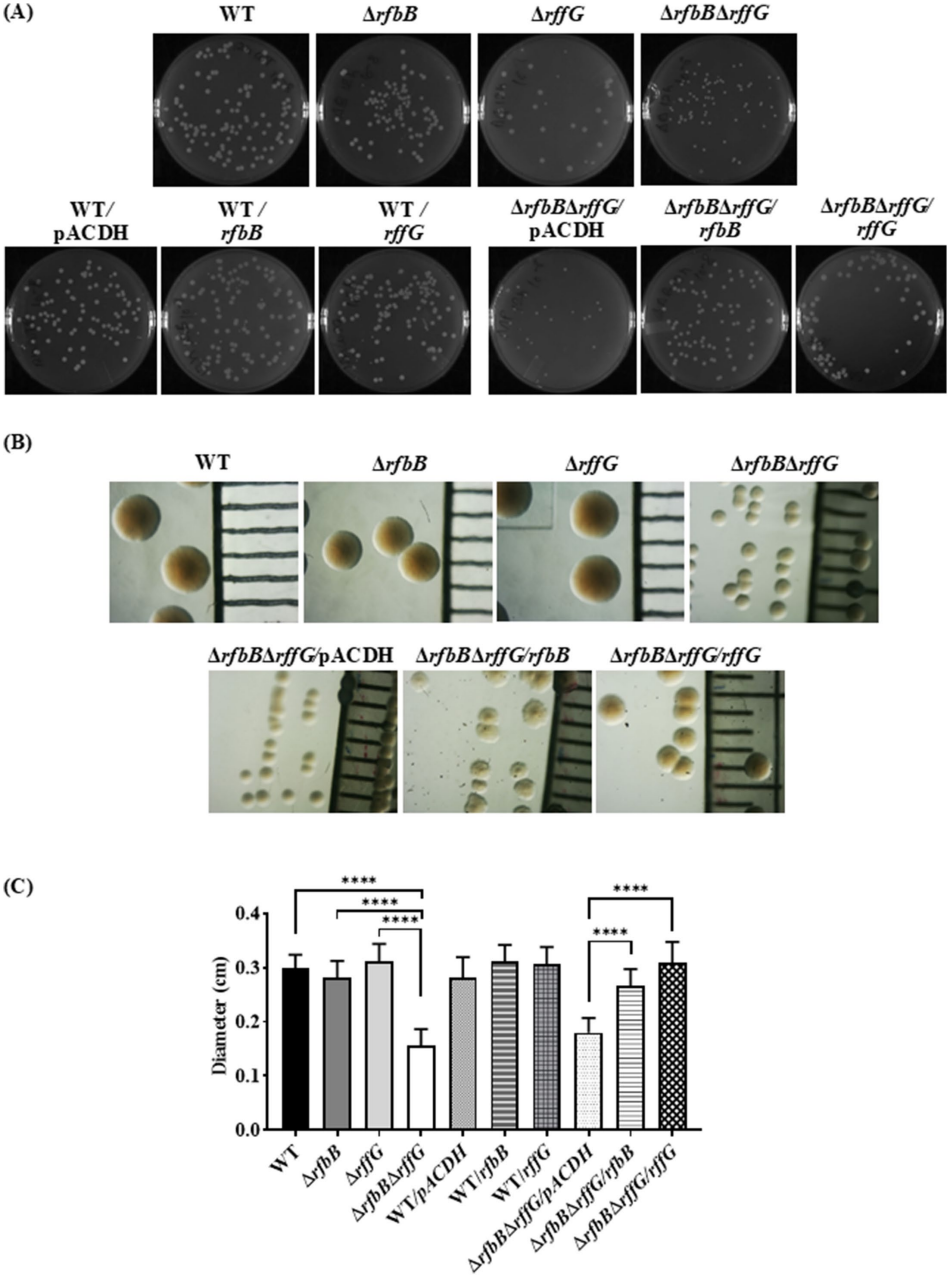
*Salmonella* Typhimurium Δ*rfbB*Δ*rffG* strain forms smaller colonies on LB agar. **(A)** *S.* Typhimurium WT, Δ*rfbB*, Δ*rffG*, Δ*rfbB*Δ*rffG* and complemented strains were plated on LB agar plates, and their images were captured after 16 h of incubation at 37°C. **(B)** Representative images depicting individual colony morphology of the above-mentioned strains obtained through a light microscope. **(C)** Diameter of the colony of WT, Δ*rfbB*, Δ*rffG* and Δ*rfbB*Δ*rffG* and the complemented strains on LB agar plates. Statistical analysis was performed using one-way ANOVA, where ****p < 0.0001. Data are representative of three independent experiments plotted as mean ± SEM. Scale Bar for **(B)**: 1 mm per unit.

Interestingly, we observed that the colonies formed by Δ*rfbB*Δ*rffG* strain on LB agar plates were considerably smaller in size when compared to the WT or the single-deletion mutants (Figure 1A). Complementing with the WT copy of either of the genes restored the colony size of the Δ*rfbB*Δ*rffG* to that of the WT (Figure 1C). This indicated that the Δ*rfbB*Δ*rffG* strain was more compromised in growth on solid media than in liquid media. Also, we noted that the colony texture of the Δ*rfbB*Δ*rffG* strain appeared much smoother compared to the WT and the single-deletion mutants (Figure 1B).

### *S.* Typhimurium Δ*rfbB*Δ*rffG* have distinct cell morphology as revealed by atomic force microscopy

**Figure 2:**
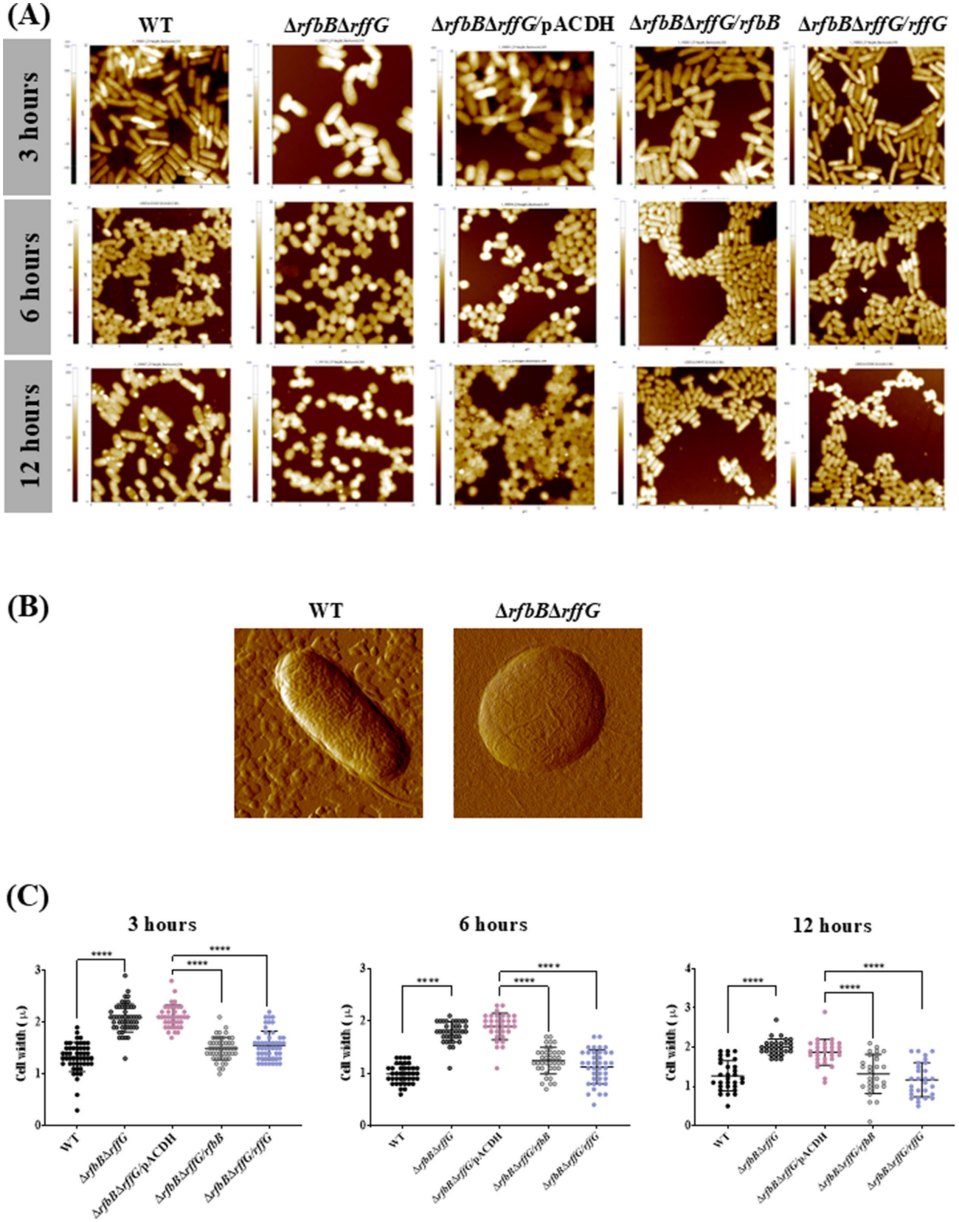
Complementation of *S.* Typhimurium Δ*rfbB*Δ*rffG* strain with WT copy of *rfbB* or *rffG* restores cellular width. **(A)** The bacterial strains were grown for the indicated time points (3, 6 or 12 h) and AFM images were acquired in the non-contact mode. **(B)** Single cell images of the WT and the Δ*rfbB*Δ*rffG* at 6 h. **(C)** Quantification of the cellular width was performed and for each condition, the width of at least 50 cells was determined. Data are representative of 3 independent experiments plotted as mean ± SEM. Statistical analysis was performed using two-way ANOVA, where * *p* < 0.05; ** *p* < 0.01; *** *p* < 0.001 and **** *p* < 0.0001.

Furthermore, differences in the cellular morphology among the different strains were examined using AFM imaging in a kinetic manner (Figure 2 and Supplementary Figure S6). The cells of the Δ*rfbB*Δ*rffG* strain were wider than those of the WT strain and single-deletion mutants. At 6 h and 12 h of growth, most of the cells of the Δ*rfbB*Δ*rffG* strain exhibited a round or spherical morphology (Figures 2A,B). Quantification of the cellular width revealed that the Δ*rfbB*Δ*rffG* strain was significantly wider than the WT strain. This phenotype was completely restored upon the expression of the WT copy of either *rfbB* or *rffG* in trans (Figure 2C).

### *S.* Typhimurium Δ*rfbB*Δ*rffG* is highly susceptible to bile stress and cell wall targeting antibiotics

**Figure 3:**
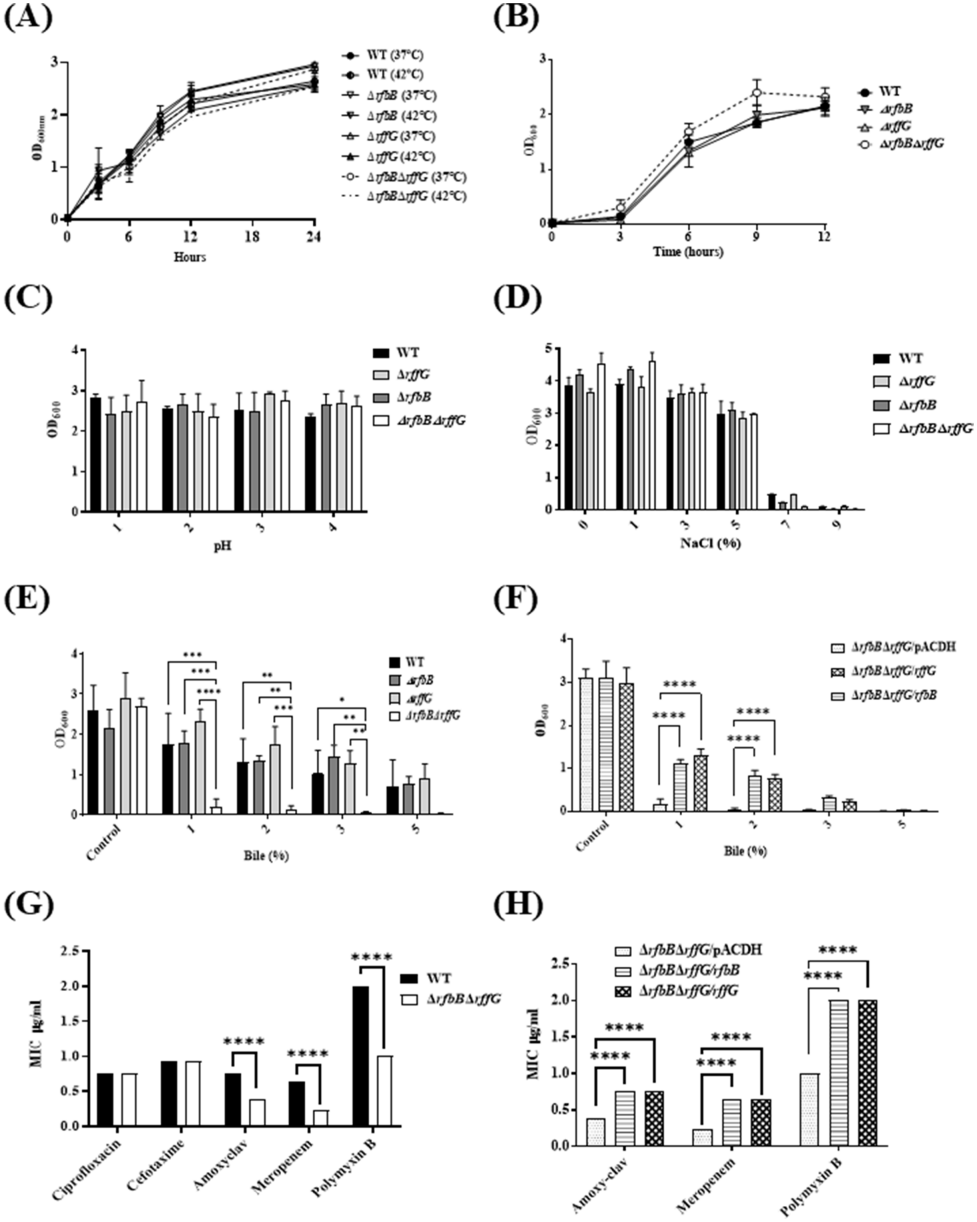
*Salmonella* Typhimurium Δ*rfbB*Δ*rffG* strain is susceptible to bile and cell wall targeting antibiotics. *S*. Typhimurium WT and Δ*rfbB*Δ*rffG* were grown **(A)** in LB at 37°C and 42°C **(B)** in minimal media. To test the effects of growth at high temperature, *S*. Typhimurium WT, Δ*rfbB*, Δ*rffG*, and Δ*rfbB*Δ*rffG* were grown in LB and media at 37°C and 42°C overnight. Post incubation, the cultures were normalized to an optical density (OD) of 2. Subsequently, 0.2% (v/v) of normalized cultures were inoculated into LB medium and incubated for 3 h. Following this, 0.2% (v/v) of the culture was transferred to respective tubes containing LB media and incubated at 37°C and 42°C with shaking at 160 RPM. Optical density measurements were recorded at specified time points. *S.* Typhimurium WT, Δ*rfbB*, Δ*rffG* and Δ*rfbB*Δ*rffG* growth in 5 mL LB broth **(C)** of different pH, and **(D)** with different concentrations of NaCl. **(E)** *S*. Typhimurium WT, Δ*rfbB*, Δ*rffG* and Δ*rfbB*Δ*rffG* and **(F)** complemented strains, Δ*rfbB*Δ*rffG*/pACDH, Δ*rfbB*Δ*rffG/rfbB*, Δ*rfbB*Δ*rffG/rffG* strains were treated with the indicated concentrations of bile and grown for 8 h at 37°C and 160 rpm. Bacterial growth was quantified by measuring OD at 600 nm. Epsilometer Test (E-Test) results to determine MIC of the **(G)** WT and the Δ*rfbB*Δ*rffG* strains as well as the **(H)** complemented strains. Briefly, overnight grown cultures were normalized to OD 2 at 600 nm and plated onto the Mueller Hinton agar plates by spread plate method. Pre-coated Ezy-MIC strips with selected antibiotics were placed in the middle of the agar plates with the help of a sterile swab and the plates were incubated at 37°C for 18 h. Data are representative of three independent experiments and plotted as mean ± SEM. Statistical analysis was performed using two-way ANOVA, where * *p* < 0.05; ** *p* < 0.01; *** *p* < 0.001 and **** *p* < 0.0001.

The susceptibility of these strains to common stresses encountered by *S.* Typhimurium was evaluated. The four strains were grown in LB at 37°C, high temperature (42°C) and minimal media at 37°C; however, no significant growth alterations among the strains were noted (Figures 3A,B). Furthermore, no differences between strains were observed in pH and osmolar stresses (Figures 3C,D). Enteric pathogens such as *S.* Typhimurium are resistant to high concentrations of bile (van Velkinburgh and Gunn, 1999); therefore, a growth susceptibility assay was conducted by exposing the strains to different concentrations of bile. No growth differences were observed between the WT and the single-deletion mutants (Figure 3E). Strikingly, the Δ*rfbB*Δ*rffG* strain exhibited a significant growth defect in a dose-dependent manner. As depicted in Figure 3F, the bile-sensitive phenotype of the Δ*rfbB*Δ*rffG* strain was rescued upon complementation with the WT copy of either *rffG* or *rfbB*.

Antimicrobial susceptibility of these strains was also tested by exposing the four strains to commonly used antibiotics, ciprofloxacin (fluroquinolone), amoxyclav, meropenem (*β*-lactam antibiotics), and polymyxin B (cationic antimicrobial polypeptide). Although there were no notable differences in the minimum inhibitory concentration (MIC) among the strains treated with ciprofloxacin, the Δ*rfbB*Δ*rffG* strain exhibited lower MIC values for amoxyclav, meropenem, and polymyxin B (Figure 3G). This phenotype could also be restored by complementing with the WT copy of either *rffG* or *rfbB* in trans (Figure 3H).

### *S.* Typhimurium Δ*rfbB*Δ*rffG* shows a truncated O-antigen profile, displays auto-aggregation behavior and binds more to NPN dye

**Figure 4:**
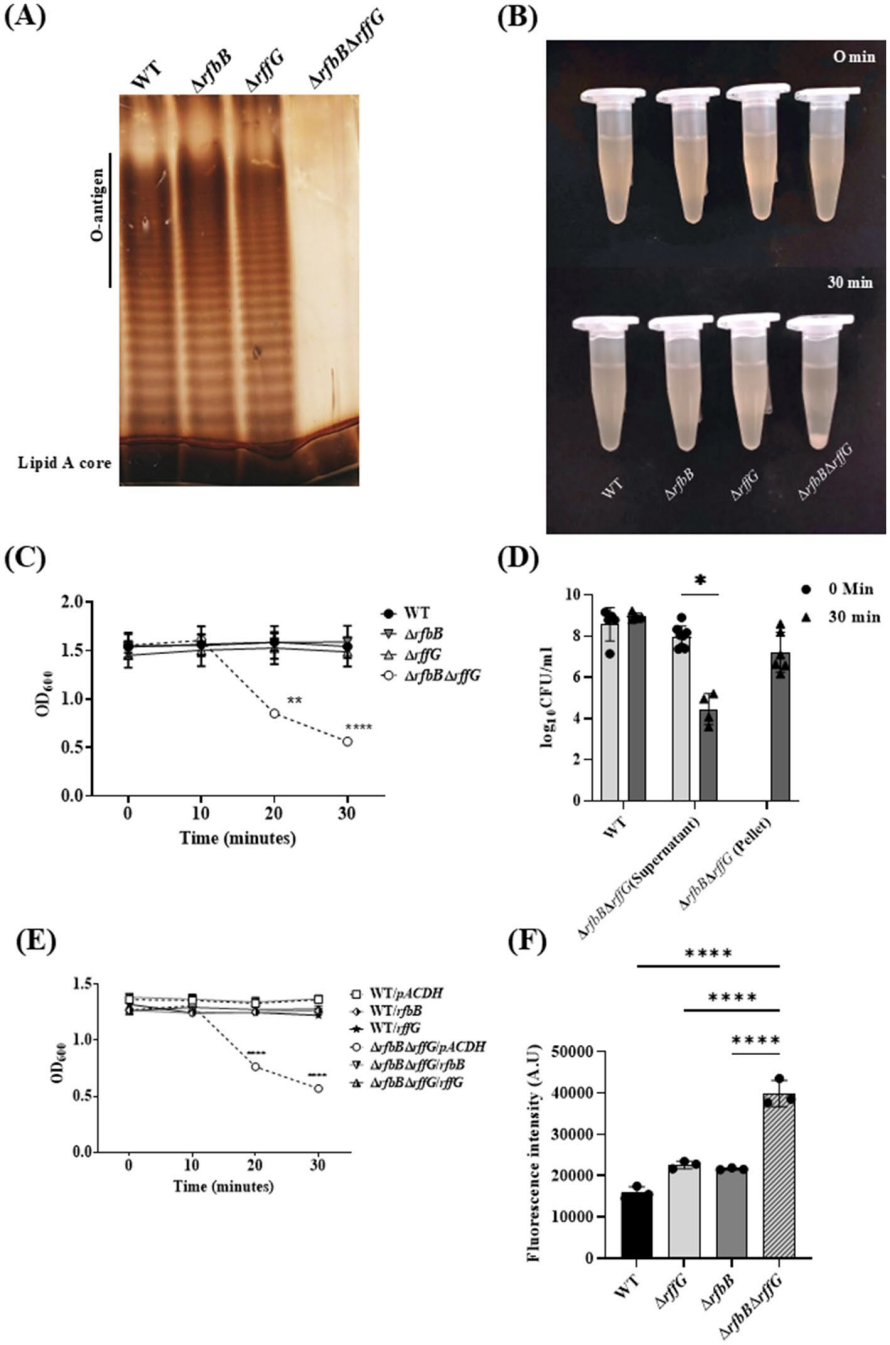
*Salmonella* Typhimurium Δ*rfbB*Δ*rffG* strain displays a truncated O-antigen profile, rapid auto-aggregation and binds more to NPN dye. **(A)** *S.* Typhimurium WT, Δ*rfbB,* Δ*rffG* and Δ*rfbB*Δ*rffG* strains were grown in LB broth at 37°C for 15 h. LPS was isolated and resolved on a 12% SDS PAGE. **(B,C)** Auto-aggregation behavior of the WT and the gene-deleted strains. Briefly, 1 mL of 1.5 OD normalized, *S.* Typhimurium WT, Δ*rfbB*, Δ*rffG* and Δ*rfbB*Δ*rffG* cultures in LB broth were kept in an upright position for 30 min. At every 10-min interval, 100 μL was aspirated from the top of the solution, and the OD was measured at 600 nm. **(D)** Post 30 min, the supernatant was aspirated from the top of the culture, and the residual pellet was resuspended in 1 mL of sterile LB. Appropriate dilutions were then plated, and colony-forming units (CFU) were recorded. **(E)** Complementation with the WT copy of the gene(s) show a rescue in the phenotype. **(F)** *S.* Typhimurium Δ*rfbB*Δ*rffG* accumulates higher amounts of the NPN dye. Briefly, the representative bacterial strains were grown to an OD of 0.5 at 600 nm. Cells were harvested, and the bacterial pellet was washed with 5 mM HEPES (pH 7.2) and adjusted to an OD of 0.5 at 600 nm. NPN was added to each well at a concentration of 10 μM. Fluorescence excitation and emission were measured at 350 nm and 420 nm, respectively. Statistical analysis for this assay was performed using one-way ANOVA, where ** *p* < 0.01. Data are representative of three independent experiments and plotted as mean ± SEM. Statistical analysis was performed using two-way ANOVA, where ** *p* < 0.01 and **** *p* < 0.0001.

The susceptibility to cell wall targeting antibiotics and bile indicated that the cell wall and membrane integrity may be compromised in the Δ*rfbB*Δ*rffG* strain. To investigate any alterations in the outer membrane protein (OMP) profile, the LPS and the OMPs of these strains were analyzed. WT and the single–deletion mutants did not display any difference in the LPS profile. However, Δ*rfbB*Δ*rffG* strain showed the absence of the repeating O-antigen structure (Figure 4A). The OMP profile of the Δ*rfbB*Δ*rffG* was also monitored and compared to the isogenic WT strain. However, no observable difference between the WT and the Δ*rfbB*Δ*rffG* strain was noted (Supplementary Figure S7). The proteins clustered around 35–45 kDa are likely to be OmpA, OmpD, and OmpC (Ray et al., 2019).

During these experiments, we also observed that the Δ*rfbB*Δ*rffG* strain quickly formed aggregates. When it was kept static at room temperature (Figures 4B,C). CFU analysis demonstrated that cell viability was unaffected during the rapid aggregation of cells (Figure 4D). This auto-aggregation behavior was optimal at 1% NaCl concentration, whereas a modulation in the concentration of NaCl (either higher or lower than 1%) reduced the auto-aggregation rate (data not shown). The auto-aggregation phenotype of the Δ*rfbB*Δ*rffG* strain could also be complemented by introducing a WT copy of either *rffG* or *rfbB* expressed in trans (Figure 4E). This auto-aggregation behavior of the Δ*rfbB*Δ*rffG* strain could be due to the absence of O-antigen repeating units which may lead to greater surface hydrophobicity as seen in higher NPN binding (Figure 4F), culminating in higher cell-to-cell aggregation.

### RNA-seq reveals differential gene expression between the WT and the Δ*rfbB*Δ*rffG* strain

A global transcriptomic analysis of 22 distinct infection-related condition in *S.* Typhimurium was published as a *Salmonella* compendium (Kröger et al., 2013). We investigated the expression of *rffG*, *rfbB* as well as other well-known genes from the *Salmonella* compendium v2.0 (Supplementary Table S3). Further, the phenotypic characterization of the *S.* Typhimurium WT, Δ*rffG*, Δ*rfbB* and Δ*rfbB*Δ*rffG* strains in this study revealed that major differences occurred only in the Δ*rfbB*Δ*rffG* strain, whereas the single-deletion strains did not display any observable differences upon comparision to the isogenic WT strain. Therefore, a transcriptomics experiment was conducted to identify differentially regulated genes in the WT and the Δ*rfbB*Δ*rffG* strains. RNA-seq analysis revealed considerable gene expression differences between the WT and the Δ*rfbB*Δ*rffG* strain. Those genes found to be significantly modulated were mapped to pathways using KEGG Mapper (Supplementary Figure S8).

Firstly, it was observed that more number of pathways were downregulated in the Δ*rfbB*Δ*rffG* strain as compared to pathways that were upregulated (Supplementary Figure S8). The genes belonging to flagellar assembly pathway as well as genes involved in the infection process, specifically invasion-related genes, were among the most significantly downregulated genes in the Δ*rfbB*Δ*rffG* strain. Other pathways which play indispensable roles during the pathogenesis of *S.* Typhimurium such as chemotaxis, quorum sensing, O-antigen biosynthesis, and LPS biosynthesis were also found to be downmodulated in the Δ*rfbB*Δ*rffG* strain as compared to the isogenic WT strain. Among the prominent pathways which were upregulated in the Δ*rfbB*Δ*rffG* strain as compared to the WT were the glycerophospholipid biosynthesis, cationic antimicrobial resistance, and nitrogen metabolism.

### The flagellar assembly pathway is downregulated and the Δ*rfbB*Δ*rffG* strain displays severe motility defects

**Figure 5:**
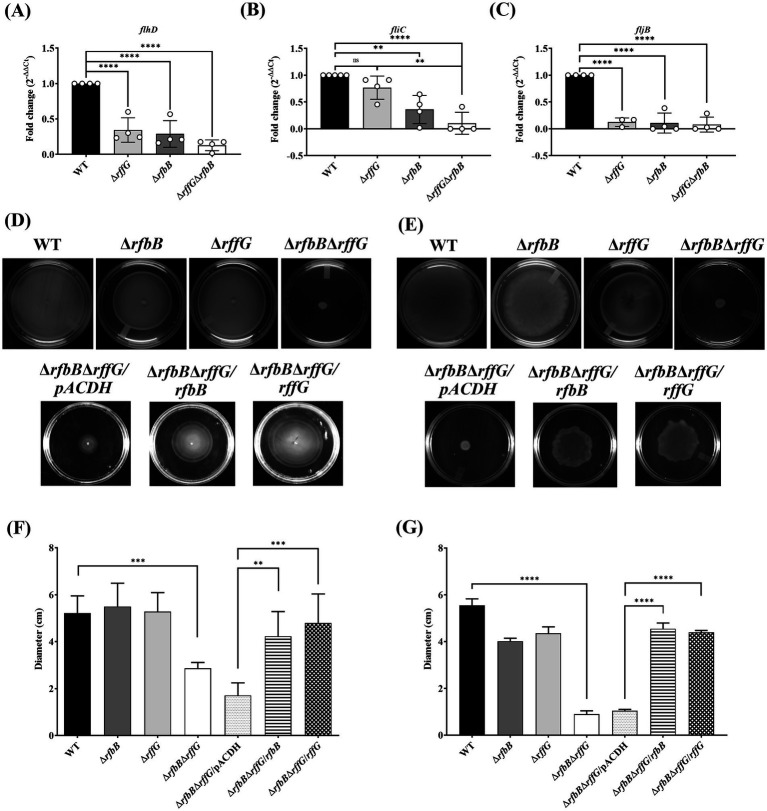
*Salmonella* Typhimurium Δ*rfbB*Δ*rffG* strain is compromised in swimming and swarming motility. Total RNA was extracted from the bacterial samples after 3 h of growth in LB broth at 37°C and 160 rpm. A qRT-PCR analysis was performed to monitor the expression of the genes, **(A)** *flhD*, **(B)** *fliC* and **(C)** *fljB* and the expression normalized with the reference gene, *gmk*. The **(D) s**wimming and **(E)** swarming motility assays were performed on 0.3 and 0.5% agar, respectively. Equal amounts of cultures were inoculated at the center of the motility-agar plates and the plates were incubated at 37°C for 8 h in an upright condition. The distance covered by each strain after 8 h post inoculation for **(F)** swimming and **(G)** swarming motility was measured and analyzed using ImageJ software. Multiple measurements were obtained for each strain. Data are representative of three independent experiments and plotted as mean ± SEM. Statistical analysis was performed using one-way ANOVA, where * *p* < 0.05; ** *p* < 0.01; *** *p* < 0.001 and **** *p* < 0.0001.

The observation from the RNA-seq was validated by focusing on the flagellar assembly pathway as well as *Salmonella* infection-related genes. The flagellar assembly pathway in *S.* Typhimurium is hierarchically organized into three classes: Class I promoter includes *flhDC* which is the master regulator, Class II and Class III gene products comprise the basal body, hook, filament, and the motor force generator, respectively, (Das et al., 2018; Ray et al., 2020). The genes, *flhD*, *fliC* and *fljB* were expressed in lower amounts in the single-deletion mutants but highly downregulated in the Δ*rfbB*Δ*rffG* strain (Figures 5A–C). However, *fliC* was not significantly downregulated in the Δ*rffG* strain.

When motility assays were performed in these strains, there were no distinguishable differences in swimming motility between the WT and the single-deletion strains (Figures 5D,F). However, the Δ*rfbB*Δ*rffG* strain was highly compromised in swimming motility and complementation with the WT copy of either *rffG* or *rfbB* in Δ*rfbB*Δ*rffG* background restored motility. The single-deletion strains appeared to be slightly but significantly compromised in swarming behavior as compared to the WT strain. The Δ*rfbB*Δ*rffG* strain, on the other hand, was found to be completely non-motile on swarming agar plates (Figures 5E,G). Complementation with the WT copy of either *rffG* or *rfbB* in Δ*rfbB*Δ*rffG* background restored the swarming defect.

### The Δ*rfbB*Δ*rffG* strain displays compromised ability to infect HeLa cells

**Figure 6:**
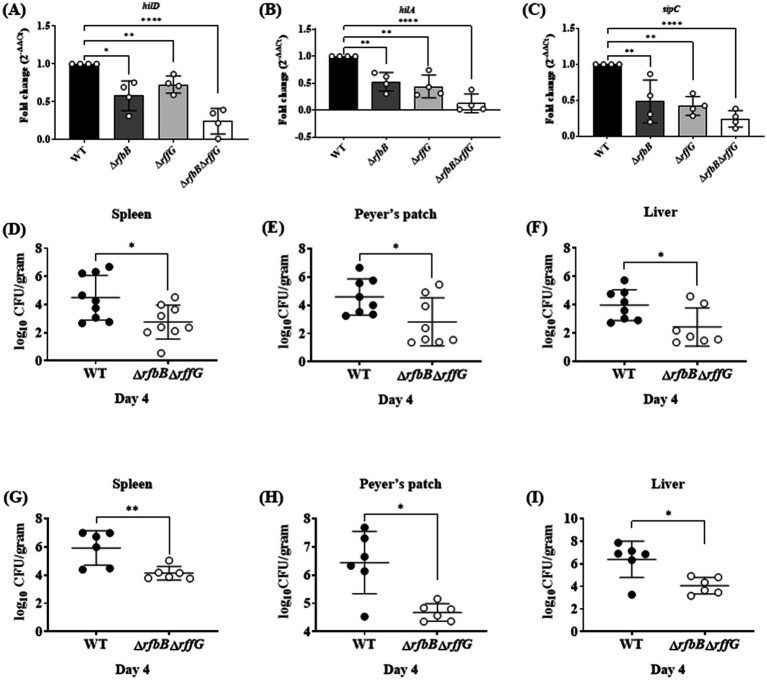
*Salmonella* Typhimurium Δ*rfbB*Δ*rffG* strain is less proficient in colonizing different organs. Total RNA was extracted from the bacterial samples after 3 h of growth in LB broth at 37°C and 160 rpm. A qRT-PCR analysis was performed to monitor the expression of the SPI-1 genes, **(A)** *hilD* **(B)** *hilA* and **(C)** *sipC,* and the expression normalized with the reference gene, *gmk.* Bacterial burden in the organs was estimated after **(D–F)** oral and **(G-I)** intraperitoneal infection of C57BL/6 mice with the WT and the Δ*rfbB*Δ*rffG* strains. Infected mice were sacrificed on day 4 post infection, organs were harvested, and the bacterial burden in the spleen, Peyer’s Patch and the liver was estimated by plating serial dilutions of the tissue homogenate on LB agar plate. Gene expression analysis data are representative of three independent experiments plotted as mean ± SEM and statistical analysis was performed using one-way ANOVA, where * *p* < 0.05; ** *p* < 0.01; *** *p* < 0.001 and **** *p* < 0.0001. For estimating bacterial burden in organs, statistical analysis was performed using the Mann Whitney U test.

*S.* Typhimurium can infect and replicate within epithelial cells and macrophages and genes present in the *Salmonella* Pathogenicity Islands 1 and 2 play key roles (Hurley et al., 2014). There are three steps in the infection process: adhesion, invasion, and intracellular replication. Initially, the expression of a few genes belonging to the SPI-1 pathway was measured. The expression of *hilA, hilD* and *sipC*, all of which regulate the expression of other key downstream effectors of the SPI-1 pathway, were downregulated in the single-deletion strains but two-fold downregulated in the Δ*rfbB*Δ*rffG* as compared to the WT strain (Figures 6A–C).

The three steps in the cellular infection process were studied in HeLa cells using the gentamicin protection assay (Supplementary Figures S9A–C). The single-deletion strains were slightly compromised than the WT in adhering to the HeLa cells, whereas the Δ*rfbB*Δ*rffG* strain was significantly compromised compared to the other strains. Additionally, the single-deletion mutants also showed a slight, although significant reduction in the number of viable bacteria at 2 h post infection compared to the WT strain. Interestingly, the Δ*rfbB*Δ*rffG* strain showed a significant reduction of the number of viable bacteria at 2 h post infection. Overall, these observations indicate that the Δ*rfbB*Δ*rffG* strain is compromised in comparison to the other strains in its ability to adhere and invade HeLa cells.

### The Δ*rfbB*Δ*rffG* strain is less proficient in colonizing different organs and induces lower pro-inflammatory cytokine response

**Figure 7:**
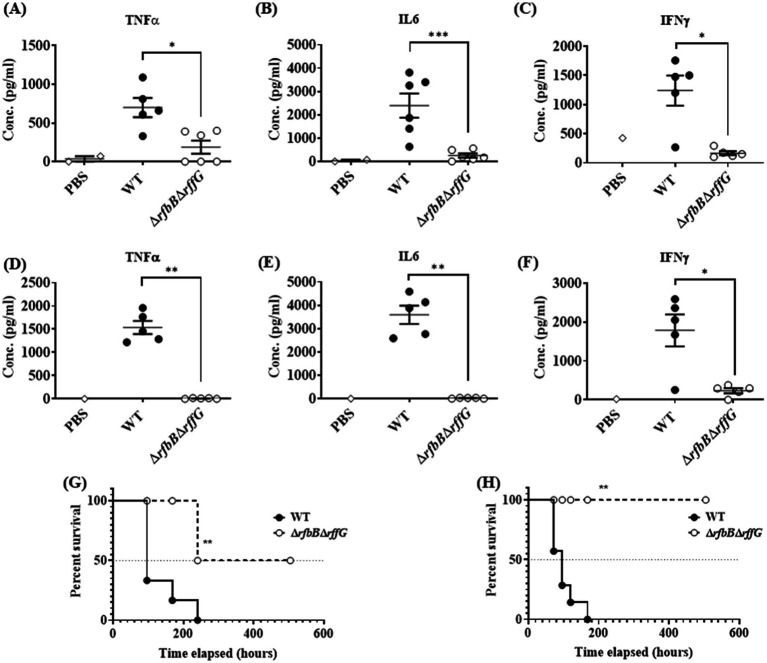
*Salmonella* Typhimurium Δ*rfbB*Δ*rffG* strain is highly attenuated in oral and intraperitoneal mice models of infection. The levels of pro-inflammatory cytokines **(A)** TNFα **(B)** IL6, and **(C)** IFNγ at day 4 post infection were estimated from the serum collected from mice infected orally with 1×10^8^ CFU of the WT and Δ*rfbB*Δ*rffG* strains. Similarly, mice infected intraperitoneally with 1×10^3^ CFU of *S.* Typhimurium WT and Δ*rfbB*Δ*rffG* were sacrificed at day 4 post infection and the serum levels of cytokines, **(D)** TNFα **(E)** IL6 and **(F)** IFNγ were quantified. Kaplan–Meier survival analysis comparing mice survival for 21 days upon infection with the WT and the Δ*rfbB*Δ*rffG* strains, infected either, **(G)** orally or **(H)** intraperitoneally. Data are representative of three independent experiments plotted as mean ± SEM. Statistical analysis was performed using Log-rank (Mantel-Cox) test, where **p < 0.005 and ***p < 0.0001. For cytokine estimation, statistical analysis was performed using Kruskal Wallis test, where *p < 0.05; **p < 0.01 and ***p < 0.001.

To determine if the Δ*rfbB*Δ*rffG* strain would exhibit any noticeable defects during *in vivo* infection. C57BL/6 mice were infected with the WT and the Δ*rfbB*Δ*rffG* strain through oral as well as intraperitoneal (i.p.) routes. Subsequently, the organ CFU burden and other responses were estimated days 4 post infection (Figures 6D–I). In the oral infection model, the pathogens must traverse the intestinal epithelial barrier, whereas in the i.p. infection model, pathogens directly enter the systemic circulation. After 4-days post oral infection, the Δ*rfbB*Δ*rffG* strain displayed two log-fold lower infection burden in the Peyer’s patches, liver, and spleen (Figures 6D–F). It is possible that the lowered CFU burden observed in the different organs might be due to the reduced expression of SPI-1 genes needed to traverse the epithelial barrier. To address this question, C57BL/6 mice were intraperitoneally infected with the WT and the Δ*rfbB*Δ*rffG* strains as intraperitoneal infection would not require active breach of the intestinal epithelial barrier. After 4 days post infection via the intraperitoneal route, the CFU burden of the Δ*rfbB*Δ*rffG* strain was two log-fold lower compared to the WT strain (Figures 6G–I). This clearly suggested that the low proficiency to infect and colonize different organs was not solely due to the reduced expression of the SPI-1 genes in the Δ*rfbB*Δ*rffG* strain but could be due to multiple and varied mechanisms. In addition, the Δ*rfbB*Δ*rffG* strain induced lesser amounts of pro-inflammatory cytokines such as TNF-*α*, IL6, and IFN-*γ* than the WT strain in both the oral (Figures 7A–C) and the i.p. (Figures 7D–F) modes of infection.

### Δ*rfbB*Δ*rffG* strain is attenuated in both oral and intraperitoneal model of *S.* Typhimurium infection

Finally, survival of the mice after oral and i.p. infection was monitored for 21 days post infection. Mice infected with the WT strain succumbed to infection as early as 4 days post infection and 100% mortality was observed by day 12. In contrast, infection with the Δ*rfbB*Δ*rffG* strain resulted in prolonged mice survival. By day 21, only 20% mortality was observed (Figure 7G). Similarly, in experiments where the mice were infected via the i.p. route, all the WT-infected mice succumbed by day 7 post infection whereas the Δ*rfbB*Δ*rffG* strain showed no mortality till 21 days post infection (Figure 7H). Overall, these results suggest that the Δ*rfbB*Δ*rffG* strain was highly attenuated in causing systemic disease in C57BL/6 mice compared to the WT strain.

## Discussion

In this study, we sought to investigate how the absence of the genes, *rffG* and *rfbB* affect the physiology of *S.* Typhimurium. Disruptions in the biosynthetic pathways of O-antigen and ECA can lead to significant cell shape abnormalities and activate stress response systems in various bacterial species. These defects primarily result due to limiting the availability of Und-P, which is important in the synthesis of key molecules (Supplementary Figure S1), leading to morphological alterations. *E.coli* (Danese et al., 1998; Jorgenson and Young, 2016; Jorgenson et al., 2016; Jorgenson and Bryant, 2021) and *S. flexneri* (Qin et al., 2025) show different tolerance to UndP sequestrations, where limited production of ECA renders *S. flexneri* more susceptible to cell death. In *E. coli*, mutations in the O-antigen flippase gene (wzxB) or ligase gene (waaL) result in the buildup of Und-PP-linked intermediates, causing cells to become swollen, filamentous, or form chains (Jorgenson and Young, 2016). Similarly, in the ECA pathway, mutations in the *wecE* and *rfbA* lead to the accumulation of ECA-lipid II intermediates, sequestering Und-P and causing cell shape defects and sensitivity to detergents (Jorgenson and Young, 2016; Jorgenson et al., 2016). In *Serratia marcescens*, mutants defective in ECA biosynthesis trigger activation of the regulator of capsule synthesis (Rcs) stress response system (Castelli and Véscovi, 2011; Castelli et al., 2008). In *Bacillus subtilis*, depletion of undecaprenyl pyrophosphate phosphatase activity leads to morphological defects and strongly activates the σM-dependent cell envelope stress response (Zhao et al., 2016). Recent studies reveal that UDP-glucose acts as a sensing molecule regulating cell size and division in *E. coli* (Hill et al., 2013). It links nutrient availability with cell division by activating OpgH, an inner-membrane glycosyltransferase involved in bacterial envelope biogenesis. In nutrient-rich conditions, UDP-glucose promotes OpgH-mediated inhibition of FtsZ ring formation by direct interaction with its N-terminal domain, delaying cell division. Also, peptidoglycan biosynthesis is important in maintaining the cellular shape in *S.* Typhimurium (van Teeseling et al., 2017).

Initial studies revealed that the Δ*rfbB*Δ*rffG* strain demonstrated a higher OD in the stationary phase as compared to the WT and the single deletion strains but did not display any difference in the CFU in stationary phase (Supplementary Figure S5). AFM studies conducted with the various strains revealed that the Δ*rfbB*Δ*rffG* strain displayed a predominantly round morphology as compared to other strains (Figure 2). Interestingly, *Salmonella* mutants lacking ECA (Ramos-Morales et al., 2003) or LPS (Spöring et al., 2018) do not show the round phenotype as noted in Δ*rfbB*Δ*rffG* strain. Our double mutants displays a change in cell morphology as it shows increased cellular width but no filamentation observed as compared to WT (Figure 2). Mutations in *mre* and *rod*, which are components of cytoskeletal arrangements, in *S.* Typhimurium give rise to a round cellular morphology (Costa and Antón, 1993). MinC and MinD are required for proper cell division. In the absence of MinC or MinD, the FtsZ ring fails to locate to the middle of a cell, leading to abnormal cell division and morphological aberrations (Wu et al., 2016). It is possible that the change in cell shape in the Δ*rfbB*Δ*rffG* strain may account for an increased OD (Yoon et al., 2015; Stevenson et al., 2016) compared to the WT or single deletion strains. Further work is required to shed more insight into the mechanisms at play that affects the distinct cellular morphology in the Δ*rfbB*Δ*rffG* strain.

We subjected these strains to various stresses that *S.* Typhimurium commonly encounters during the host colonization process. Although no growth difference was detected among the strains in many stress conditions, the Δ*rfbB*Δ*rffG* strain was found to be highly susceptible to bile-induced stress, with, cell wall targeting antibiotics and cationic polypeptides (Figure 3). LPS acts as a membrane barrier; consequently, LPS mutants are susceptible to bile (Urdaneta and Casadesús, 2017). *Salmonella* strains lacking ECA do not show alterations in morphology, LPS profile or motility, although they are attenuated in mice models of infection (Gilbreath et al., 2012). The outer membrane of Gram-negative bacteria is unique among all biological membranes by virtue of their properties to exclude hydrophobic substances, primarily due to LPS. The probe 1-N-phenylnaphthylamine (NPN) is used to study membranes as it gives strong fluorescence in phospholipid environments but fluoresces weakly in aqueous environments (Helander and Mattila-Sandholm, 2000). The dye accumulated to a similar extent in the WT and the single deletion strains. However, an increased amount of fluorescence was observed in the Δ*rfbB*Δ*rffG* strain (Figure 4F), indicating enhanced surface hydrophobicity which may be responsible for greater aggregation (Figures 4B–E). In fact, the susceptibility to cell wall targeting antibiotics and bile indicated that the outer membrane integrity may be compromised in the Δ*rfbB*Δ*rffG* strain (Figures 3E–H).

A comparative transcriptomic analysis (RNA-seq) between the WT and the Δ*rfbB*Δ*rffG* strains showed major alterations in several pathways: motility, invasion, chemotaxis (Supplementary Figure S8). The flagellar assembly pathway was one, among the many prominent pathways that was found to be highly downregulated in the Δ*rfbB*Δ*rffG* strain. In fact, motility studies revealed that the Δ*rfbB*Δ*rffG* strain was significantly compromised in swimming as well as swarming motility as compared to the WT and the single deletion strains (Figure 5). Swimming motility represents individual movement in liquid environments using flagella, whereas swarming motility is also flagella-driven, multicellular and on solid or semi-solid surfaces (Thakur et al., 2020). Mutations in genes of the O antigen and ECA biosynthesis pathways can activate the Rcs phosphorelay system, a key envelope stress response mechanism in bacteria belonging to the Enterobacteriaceae family (Meng et al., 2021; Liu et al., 2022). Sequestration of Und-P in O-antigen and ECA mutants due to the accumulated biosynthetic intermediates compromises peptidoglycan synthesis and cell wall integrity, leading to morphological defects and triggering stress responses, including the Rcs system (Castelli and Véscovi, 2011; Spöring et al., 2018; Castelli et al., 2008). The Rcs phosphorelay is a critical envelope stress-sensing signal transduction pathway that plays a major role in motility. Through its transcriptional regulator RcsB, the phosphorelay directly represses flhDC expression, which encodes the master transcriptional regulator, FlhD4C2. Reduced flhDC levels leads to decreased flagellum production and inhibits motility (Francez-Charlot et al., 2003). In *E. coli*, the Rcs two component signaling pathway plays a role in repression of motility in LPS mutants (Girgis et al., 2007). In *S.*, Typhimurium Rcs activation represses flagellar gene expression via flhDC, leading to impaired swarming motility (Wang et al., 2007). Similarly, in *E. coli*, the RcsC-YojN-RcsB phosphorelay modulates capsular synthesis and swarming behavior (Takeda et al., 2001). The lack of proper LPS perturbs the outer membrane, leading to activation of signaling pathways, including RpoE and Rcs phosphor relay system, and degradation of FlhDC, the class I motility regulator (Spöring et al., 2018). In *Proteus mirabilis*, disruption of cell envelope-associated genes impairs swarmer cell development and motility through various mechanisms (Morgenstein and Rather, 2011). RcsB also promotes the expression of several fimbrial genes, including paralogs of the fimbrial transcriptional regulator MrpJ (Bode et al., 2015). Collectively, these findings reinforce the role of the Rcs system in coordinating bacterial envelope stress responses with motility regulation. It is possible that in the Δ*rfbB*Δ*rffG* strain, the absence of the O-antigen and ECA leads to loss of surface lubrication and aberrant signaling events, leading to compromised motility (Figure 5). Further studies are required to investigate the Rcs activation and other pathways to understand their functional roles in these mutants strains.

Another, pathway that was found to be highly downmodulated in the Δ*rfbB*Δ*rffG* strain was the SPI-1 pathway (Supplementary Figure S8 and Figure 6). The significant difference in the invasion for the Δ*rfbB*Δ*rffG* strain observed in HeLa, could be due to less SPI-1 effector proteins (Supplementary Figure S9). Both SPI-1 and the flagellar biosynthesis pathway are essential for successful colonization of mice (Dieye et al., 2009; Stecher et al., 2004). Studies have shown that *inv* mutants of *S.* Typhimurium, which do not have functional SPI-1, when administered perorally to BALB/c mice, results in higher 50% lethal doses (LD_50_). In addition, *inv* mutants were compromised in their ability to colonize the Peyer’s patches, small intestinal wall, and the spleen when administered perorally (Porter and Curtiss, 1997). However, no such differences were observed when these strains were administered intraperitoneally. Although the Δ*rfbB*Δ*rffG* strain had the ability to colonize different organs, it was less proficient than the parent, WT strain (Figure 6) and induced lower pro-inflammatory cytokine responses (Figure 7). Importantly, the Δ*rfbB*Δ*rffG* strain did not cause lethal infection when administered via oral as well as the i.p. routes of infection (Huang et al., 2016; Zhou et al., 2023). This observation is consistent with previous findings in *Candida albicans*, where it was observed that a GAL102p mutant (a homolog of dTDP-glucose 4,6-dehydratase) was unable to grow in resident peritoneal macrophages. The mutant also elicited a weak pro-inflammatory cytokine response *in vitro* as well as in an *in vivo* mouse model of systemic candidiasis (Sen et al., 2011). Both O-antigen, ECA and the flagella are important antigens recognized by the immune system (Hajam et al., 2017; Kintz et al., 2017). The absence or down regulation of these components would cause aberrant immune responses in mice infected with the Δ*rfbB*Δ*rffG* strain. Earlier studies have also reported that the LPS or ECA mutant are highly attenuated in *S.* Typhimurium oral infection model (Kong et al., 2011; Gilbreath et al., 2012). The absence of the O-antigen and ECA in the Δ*rfbB*Δ*rffG* strain may make the infection cycle non-optimal and/or these strains may be more susceptible to complement mediated lysis (Murray et al., 2006). Despite the lowered virulence of the Δ*rfbB*Δ*rffG* strain, its ability to grow within host organs may be a concern for its proposed use as a live attenuated vaccine (Huang et al., 2016). Previously, we have demonstrated that another attenuated strain, Δ*rpoS*, can colonize host organs (Rananware et al., 2021). It is likely that mice can co-exist or eventually clear the infection when infected with attenuated *S.* Typhimurium strains.

**Figure 8:**
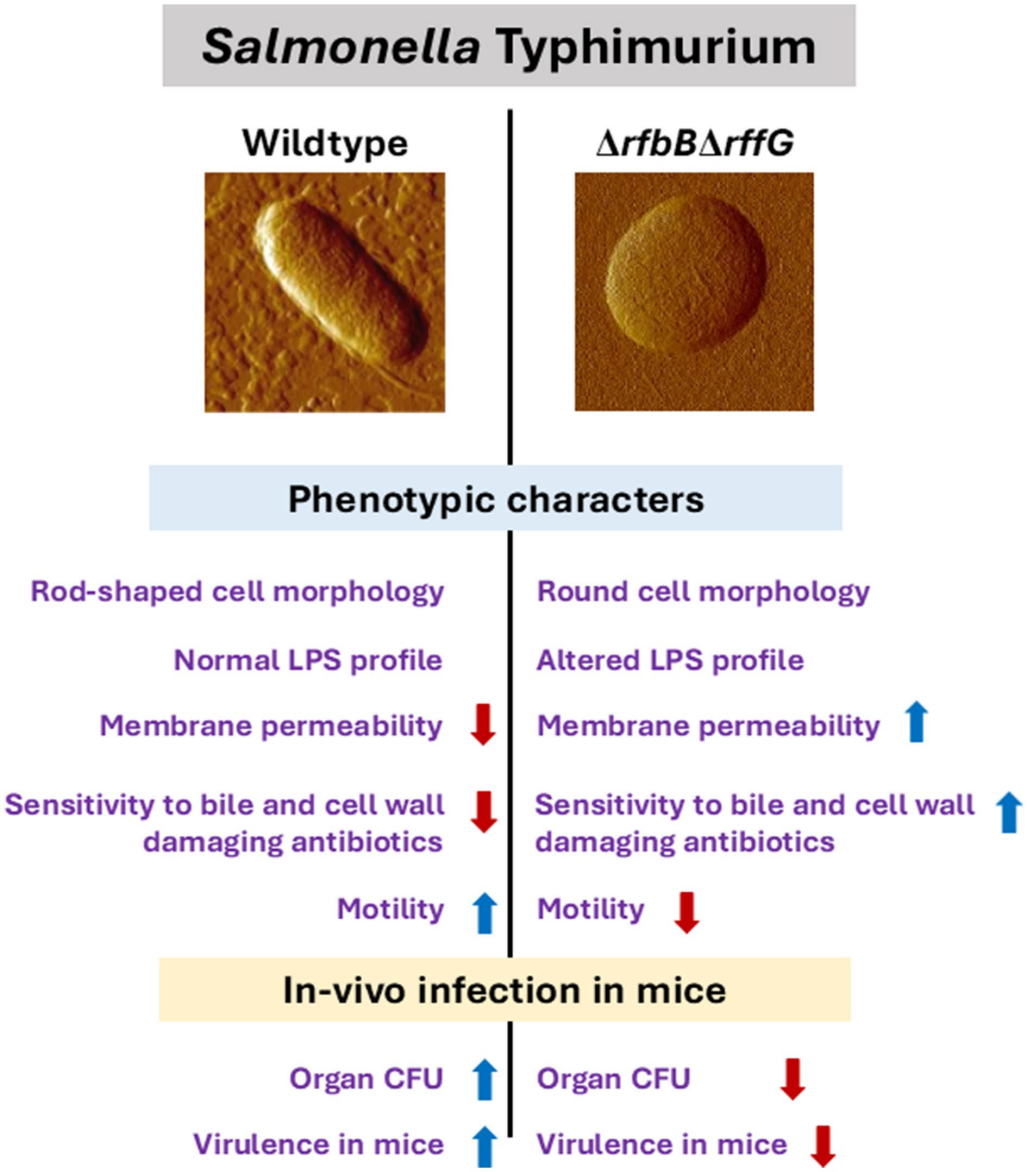
*Salmonella* Typhimurium WT and ∆*rfbB*∆*rffG* show distinct physiological and phenotypic differences. The genes, *rfbB* and *rffG,* encode the enzyme dTDP-glucose 4,6- dehydratase, which catalyzes intermittent steps of O-antigen and ECA biosynthesis. Functional loss of dTDP-glucose 4,6-dehydratase renders *S.* Typhimurium strains incapable of synthesizing both O-antigen and ECA. This leads to profound physiological differences between the *S.* Typhimurium, WT and the Δ*rfbB*Δ*rffG* strains. The Δ*rfbB*Δ*rffG* strain exhibits distinct cellular morphology, altered LPS profile, increased outer membrane permeability and susceptibility to bile and cell wall targeting antibiotics. Functional loss of dTDP-glucose 4,6-dehydratase also led to inhibition of motility and a reduced ability to colonize different organs in the mouse model of infection. Consequently, the Δ*rfbB*Δ*rffG* strain displayed significant virulence attenuation as compared to the WT.

Finally, we present a model that accounts for the key observations (Figure 8). Briefly, the genes, *rffG* and *rfbB* are paralogs that encode the enzyme dTDP-glucose 4,6- dehydratase, which is known to catalyze steps of O-antigen and ECA biosynthesis (Supplementary Figure S1). Consequently, the functional loss of dTDP-glucose 4,6-dehydratase activity leads to profound physiological differences, a reduced ability to colonize different organs and lowered virulence in the mouse model of infection. The LPS biosynthesis pathway has generated a lot of optimism as a drug target (Zhang et al., 2018). To the best of our knowledge, this is one of the primary reports to demonstrate in detail the physiological consequences of the loss of dTDP-glucose 4,6- dehydratase activity in *S.* Typhimurium. This study, therefore, lays the foundation for further research and better understanding of the combinatorial roles of O-antigen and ECA, either directly or indirectly, on *S.* Typhimurium physiology and pathogenesis.

## Data Availability

The RNAseq dataset accession number presented in this article is not readily available because the experiment was performed with one biological replicate to obtain a preliminary idea regarding the DEGs in the Δ*rfbB*Δ*rffG* strain compared to the isogenic WT control after growth in a nutrient rich medium (LB) at 3 hours. The findings from this preliminary analysis were further validated using qPCR (Figure 5 and Figure 6). Requests to access this dataset and all other data should be directed to Dipankar Nandi (nandi@iisc.ac.in).
