# Supplementary material for: Functional loss of rffG and rfbB, encoding dTDP-glucose 4,6-dehydratase, alters colony morphology, cell shape, motility and virulence in Salmonella Typhimurium
Source: Front Microbiol. 2025 May 21;16:1572117. doi: 10.3389/fmicb.2025.1572117 (PMC12136496; doi:10.3389/fmicb.2025.1572117)
Supplement: Supplementary file 1 [file Data_Sheet_1.docx]

**SUPPLEMENTARY INFORMATION**

**Functional loss of *rffG* and *rfbB,* encoding dTDP-glucose 4,6-dehydratase, alters colony morphology, cell shape, motility and virulence in *Salmonella* Typhimurium**

Subhashish Chakraborty^1†^, Pip Banerjee^1†^, Joel P. Joseph^2^, Sanmoy Pathak^1^, Taru Verma^2^, Aagosh Kishor Karhale^1^, Deepti Chandra^1^, Mrinmoy Das^1^, Pritam Saha^1^ and Dipankar Nandi^1*^

^1^Department of Biochemistry, Indian Institute of Science, Bangalore, India

^2^Department of Bioengineering, Indian Institute of Science, Bangalore, India

^†^These authors contributed equally to this work and shares first authorship

^*^Corresponding author (nandi@iisc.ac.in/+91-80-22933051)

**Table S1. List of bacterial strains used in this study**

| **Sl. No.** | **Name of the strain** | **Source** |
| --- | --- | --- |
| 1. | *Salmonella* Typhimurium 14028s | (Allam et al., 2011) |
| 2. | *Salmonella* Typhimurium Δ*rfbB* | This study |
| 3. | *Salmonella* Typhimurium Δ*rffG* | This study |
| 4. | *Salmonella* Typhimurium Δ*rfbB*Δ*rffG* | This study |
| 5. | *Salmonella* Typhimurium WT/pACDH | This study |
| 6. | *Salmonella* Typhimurium WT/*rfbB* | This study |
| 7. | *Salmonella* Typhimurium WT/*rffG* | This study |
| 8. | *Salmonella* Typhimurium Δ*rfbB*Δ*rffG*/pACDH | This study |
| 9. | *Salmonella* Typhimurium Δ*rfbB*Δ*rffG/rfbB* | This study |
| 10. | *Salmonella* Typhimurium Δ*rfbB*Δ*rffG/rffG* | This study |

**Table S2. List of primers used in this study**

| **Oligonucleotides (5’ 🡪 3’)** | |
| --- | --- |
| **Knockout generation primers** | |
| ***rffG:: Kan^r^*** | \| **FP** \| **AAGGAGTCTGGCGCTGATGAAACGCATTCTGGTGACCGGCGTGTAGGCTGGAGCTGCTT** \| \| --- \| --- \| \| **RP** \| **TTAGCGTTTCAGTCCTAAGCGTTCGCCCTGATAACTGCCAGTCCATATGAATATCCTCCTTAG** \| |
| ***rfbB:: Chl^r^*** | \| **FP** \| **ATGGAATAGAAAAGTGAAGATACTTATTACTGGCGGGGCAATGTAGGCTGGAGCTGCTTCG** \| \| --- \| --- \| \| **RP** \| **TTACTGGCGTCCTTCATAGTTCTGTTCTATCCAACTCTGACGGCTGACATGGGAATTAGC** \| |
| **Knockout confirmation primers** | |
| ***rffG*** | \| **FP** \| **GTCGAACCGAATATCCGTCAG** \| \| --- \| --- \| \| **RP** \| **CCAGACAGGCAATTTTGAAGC** \| |
| ***rfbB***  **(Chl^r^ internal primers)** | \| **FP** \| **GTGGTATTCACTCCAGAGC** \| \| --- \| --- \| \| **RP** \| **CCGTTGATATATCCCAATGGC** \| |
| **Gene cloning primers** | |
| ***rffG*** | \| **FP** \| **CATGCCATGGATGAAACGCATTCTGGTGACC** \| \| --- \| --- \| \| **RP** \| **CCCAAGCTTTTAGCGTTTCAGTCCTAAGCG** \| |
| ***rfbB*** | \| **FP** \| **CATGCCATGGGTGAAGATACTTATTACTGGCG** \| \| --- \| --- \| \| **RP** \| **CCCAAGCTTTTACTGGCGTCCTTCATAGTTC** \| |

| **Oligonucleotides (5’ 🡪 3’)** | |
| --- | --- |
| **Gene sequencing primers** | |
| ***rffG*** | |
| ***Fragment 1*** | \| **FP** \| **CACAGGAAACAGCTATGACC** \| \| --- \| --- \| \| **RP** \| **GCGTAGGCAGACCGTAGGTA** \| |
| ***Fragment 2*** | \| **FP** \| **TTCCGCTTCCACCATATCTC** \| \| --- \| --- \| \| **RP** \| **GCGTAGGCAGACCGTAGGTA** \| |
| ***Fragment 3*** | \| **FP** \| **TTCCGCTTCCACCATATCTC** \| \| --- \| --- \| \| **RP** \| **GTGCCAACATAGTAAGCCAG** \| |
| ***rfbB*** | |
| ***Fragment 1*** | \| **FP** \| **CACAGGAAACAGCTATGACC** \| \| --- \| --- \| \| **RP** \| **GATCCGCGACATAAGTG** \| |
| ***Fragment 2*** | \| **FP** \| **GGTGATGCATTTGGC** \| \| --- \| --- \| \| **RP** \| **GTGCCAACATAGTAAGCCAG** \| |

| **Oligonucleotides (5’ 🡪 3’)** | |
| --- | --- |
| **qPCR primers** | |
| ***hilA*** | \| **FP** \| **TAATCGTCCGGTCGTAGTGG** \| \| --- \| --- \| \| **RP** \| **TGCGGCAGTTCTTCGTAATG** \| |
| ***hilD*** | \| **FP** \| **AACGTGACGCTTGAAGAGGT** \| \| --- \| --- \| \| **RP** \| **GAACGCCGTTTTCAGATGTT** \| |
| ***sipC*** | \| **FP** \| **CGCGAATACGTTAATGCTGA** \| \| --- \| --- \| \| **RP** \| **CGCGCTCTGGGAAATACTAC** \| |
| ***flhD*** | \| **FP** \| **ATCGTCCAGGACAAAGCATC** \| \| --- \| --- \| \| **RP** \| **TCGTCCACTTCATTGAGCAG** \| |
| ***fliC*** | \| **FP** \| **TGACAGCAGCAGGTGTTACC** \| \| --- \| --- \| \| **RP** \| **CGCCACCCAGTTTGTTTAGT** \| |
| ***fljB*** | \| **FP** \| **GCCAACGACGGTGAAACTAT** \| \| --- \| --- \| \| **RP** \| **CACCCGTAGCCGCTTTAATA** \| |
| ***gmk*** | \| **FP** \| **TTCCGTTTCACATACCACGC** \| \| --- \| --- \| \| **RP** \| **CCTGCCAGTCGATATCCAGA** \| |

**Table S3. Intra-strain relative expression levels of *rffG*, *rfbB, hilA, hilD* and *ssaG* genes in *S.* Typhimurium D23580 strain (SalCom V2.0)**

| **Condition** | ***rffG*** | ***rfbB*** | ***hilA*** | ***hilD*** | ***ssaG*** |
| --- | --- | --- | --- | --- | --- |
| **EEP** | 1 | 1 | 1 | 1 | 1 |
| **MEP** | 0.88 | 0.71 | 0.95 | 0.79 | 1 |
| **LEP** | 0.78 | 0.42 | 16.41 | 12.57 | 1 |
| **ESP** | 0.64 | 0.28 | 18.64 | 11.38 | 13.34 |
| **LSP** | 0.17 | 0.04 | 0.95 | 1.01 | 1.79 |
| **MEP** | 1 | 1 | 1 | 1 | 1 |
| **NaCl shock** | 0.3 | 0.25 | 1 | 2.73 | 1 |
| **Bile shock** | 1.63 | 1.32 | 1 | 0.9 | 1 |
| **Low Fe2+ shock** | 0.92 | 0.37 | 1 | 1.13 | 1 |
| **Anaerobic shock** | 1 | 0.18 | 1.06 | 4.67 | 1 |
| **Anaerobic growth** | 1 | 1 | 1 | 1 | 1 |
| **Oxygen shock** | 1.84 | 1.45 | 3.92 | 1.56 | 1 |
| **InSPI2** | 1 | 1 | 1 | 1 | 1 |
| **Peroxide shock (InSPI2)** | 0.26 | 0.17 | 1 | 0.32 | 0.57 |
| **Nitric Oxide shock (InSPI2)** | 0.78 | 0.43 | 1 | 0.62 | 0.89 |
| **NonSPI2** | 1 | 1 | 1 | 1 | 1 |
| **InSPI2** | 0.79 | 0.6 | 0.72 | 0.97 | 136.47 |
| **ESP** | 1 | 1 | 1 | 1 | 1 |
| **Macrophage** | 1.55 | 1.2 | 0.05 | 0.2 | 10.07 |

**Note:** The genes, *hilA* and *hilD* have been tabulated as reference genes from the SPI-1 island and *ssaG* as a reference gene from the SPI-2 island. Rows (conditions) shaded in grey are the reference conditions to normalise the expression of the genes under the subsequent conditions (rows).

EEP: Early exponential phase; MEP: Mid exponential phase; LEP: Late exponential phase; ESP: Early stationary phase; LSP: Late stationary phase; Non SPI2: PCN medium (pH 7.4, 25 mM P_i_); InSPI2: PCN medium (pH 5.8, 0.4 mM P_i_).

Source: (Canals et al., 2019)


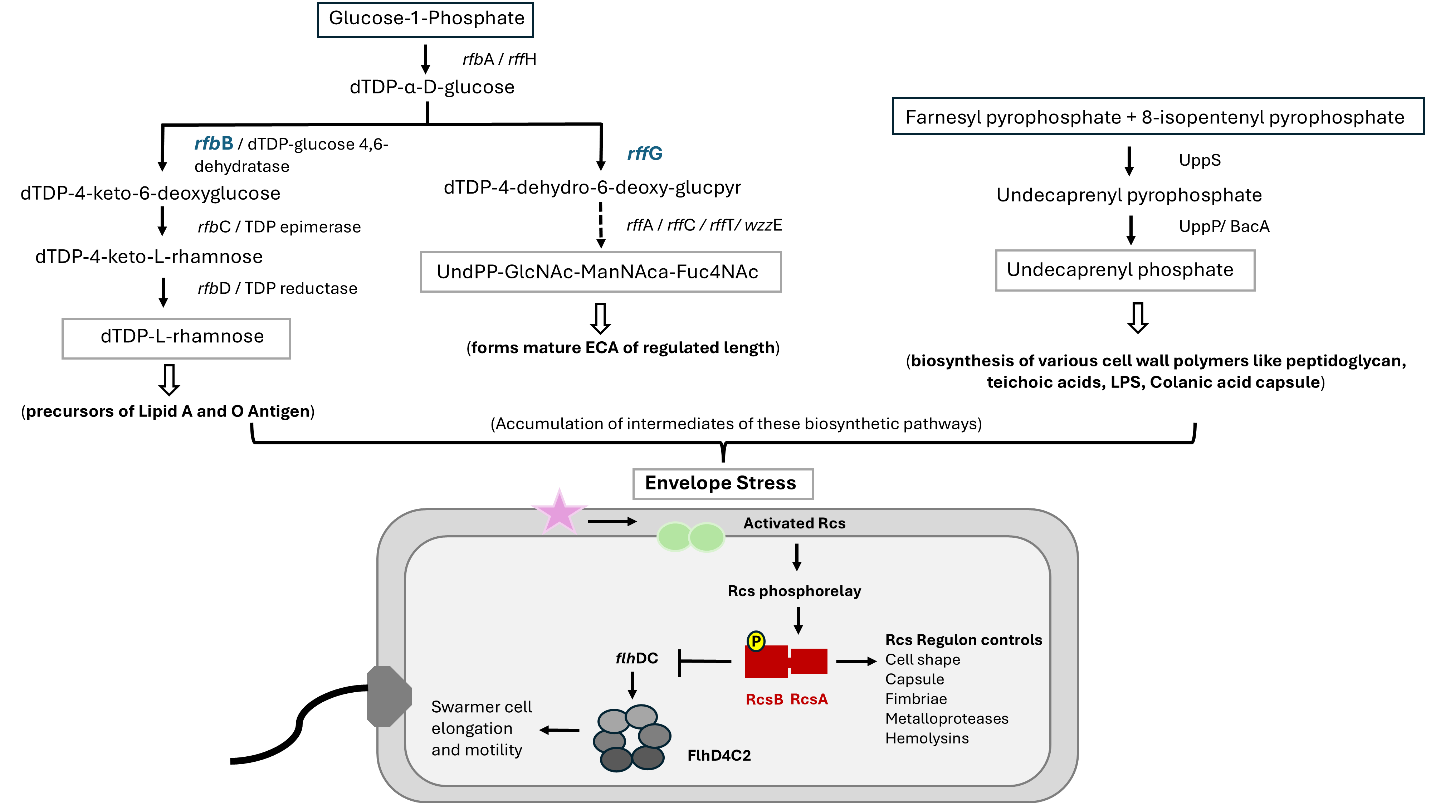


**Figure S1. Schematic depicting the roles of RfbB and RffG in various biosynthetic pathways.** RfbB and RffG catalyze the conversion of dTDP-D-glucose into TDP-4-keto-6-deoxy-D-glucose and dTDP-4-dehydro-6-deoxy-glucopyranose, respectively (Marolda et al., 1995, Parakkottil et al., 2010). The intermediate 4-keto-6-deoxy-D-glucose is further converted into L-rhamnose, a key precursor for the biosynthesis of lipid A, the core, and the O-antigen repeating units (Kong et al., 2011). Meanwhile, UndPP-GlcNAc-ManNAcA-Fuc4NAc is involved in the biogenesis of enterobacterial common antigen (ECA) (Marolda et al., 1995). Therefore, RfbB and RffG play essential roles in the biosynthesis of O-antigen and ECA, respectively. Und-P is a critical lipid carrier required for the biosynthesis of peptidoglycan, colanic acid capsule, O-antigen, and ECA (Bouhss et al., 2008). Disruptions in any of these pathways can triggering envelope stress (depicted by pink star) through the activation of the Rcs phosphorelay system (shown as green spheres) (Castelli et al., 2010, Liu et al., 2022). Activation of the Rcs system represses motility and alters cellular morphology, including changes in shape, capsule production, and fimbriae expression (Little et al., 2018). The model illustrates that the disruptions/ accumulation of intermediates in the biosynthetic pathways of L-rhamnose, UndPP-GlcNAc-ManNAcA-Fuc4NAc, and undecaprenyl phosphate (Und-P) induces envelope stress and cellular morphology.


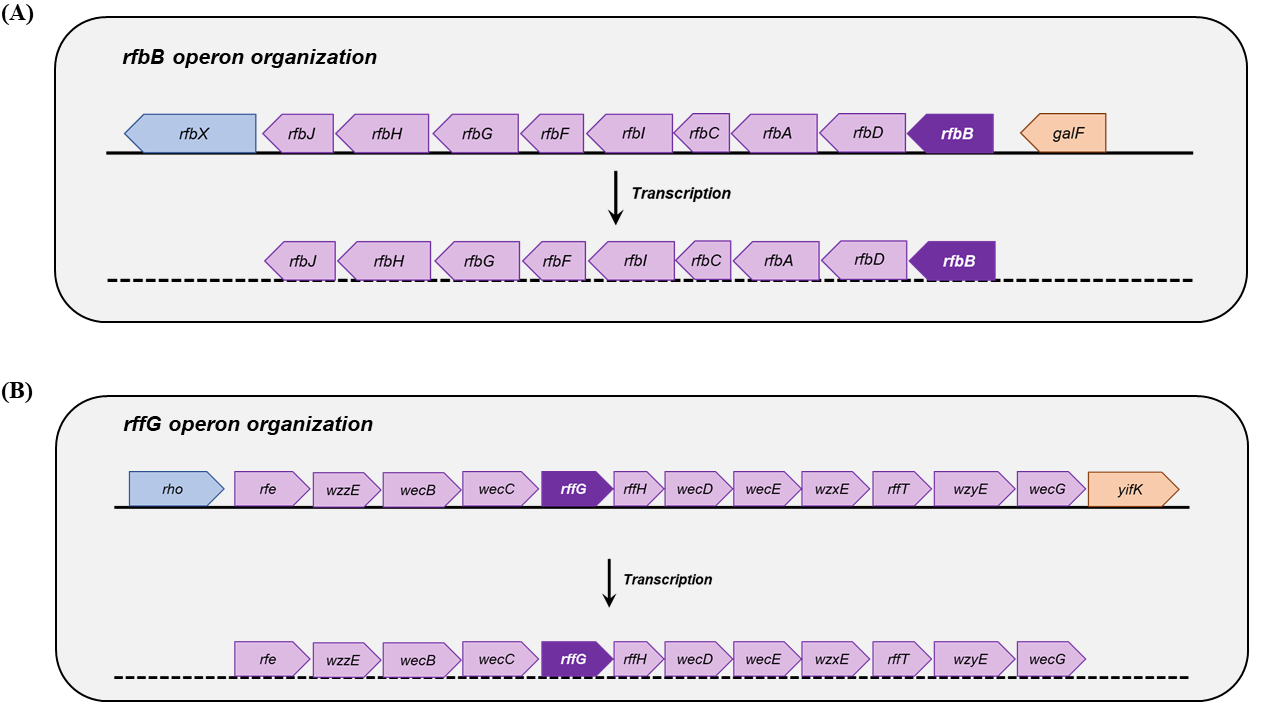


**Figure S2.** **Operon organization of *rffG* and *rfbB* in *S.* Typhimurium LT2 genome.** The genomic location and organization of the genes, **(A)** *rfbB* and **(B)** *rffG* was explored in the BioCyc database. *Salmonella* Typhimurium LT2 was selected as the query organism for this analysis. BioCyc.org is a microbial genome web portal that combines several genomes with additional information inferred by computer programs, imported from other databases and curated from biomedical literature by biologist curators. BioCyc also provides an extensive range of query tools, visualization services and analysis software (Karp et al., 2019).

##
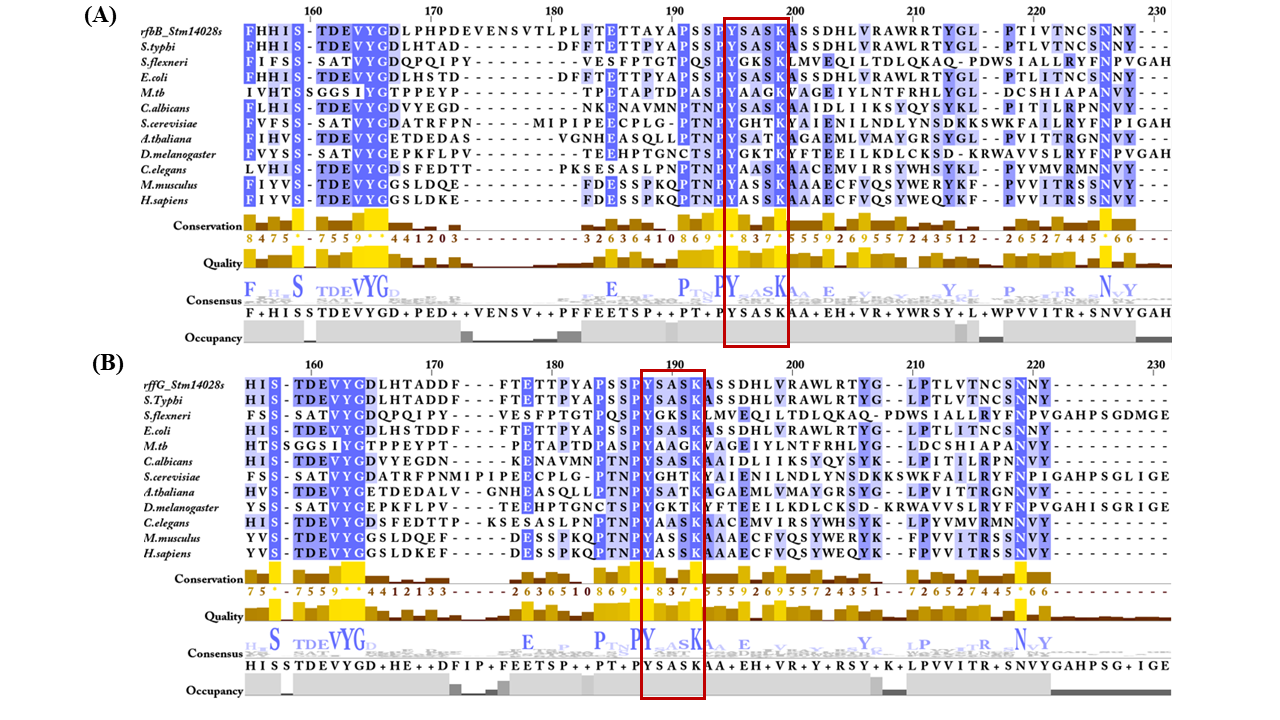


**Figure S3.** **Multiple sequence alignment (MSA) of homologs with highest percentage identity to *S.* Typhimurium 14028s encoded proteins, RfbB and RffG.** A MSA was performed using CLUSTAL Omega program and represented in JalView with homologs of **(A)** RfbB and **(B)** RffG proteins encoding the enzyme, dTDP glucose 4,6-dehydratase in *S*. Typhimurium 14028s. The reference genomes from these representative organisms have been used for performing this analysis: *Salmonella enterica subsp. enterica serovar* Typhi str*.* CT18, *Shigella flexneri*, *Escherichia coli*, *Mycobacterium tuberculosis*, *Candida albicans,* *Saccharomyces cerevisiae*, *Arabidopsis thaliana, Drosophila melanogaster, Caenorhabditis elegans, Mus musculus and Homo sapiens.* The conserved catalytic motif YXXXK in these proteins have been highlighted in the red box.


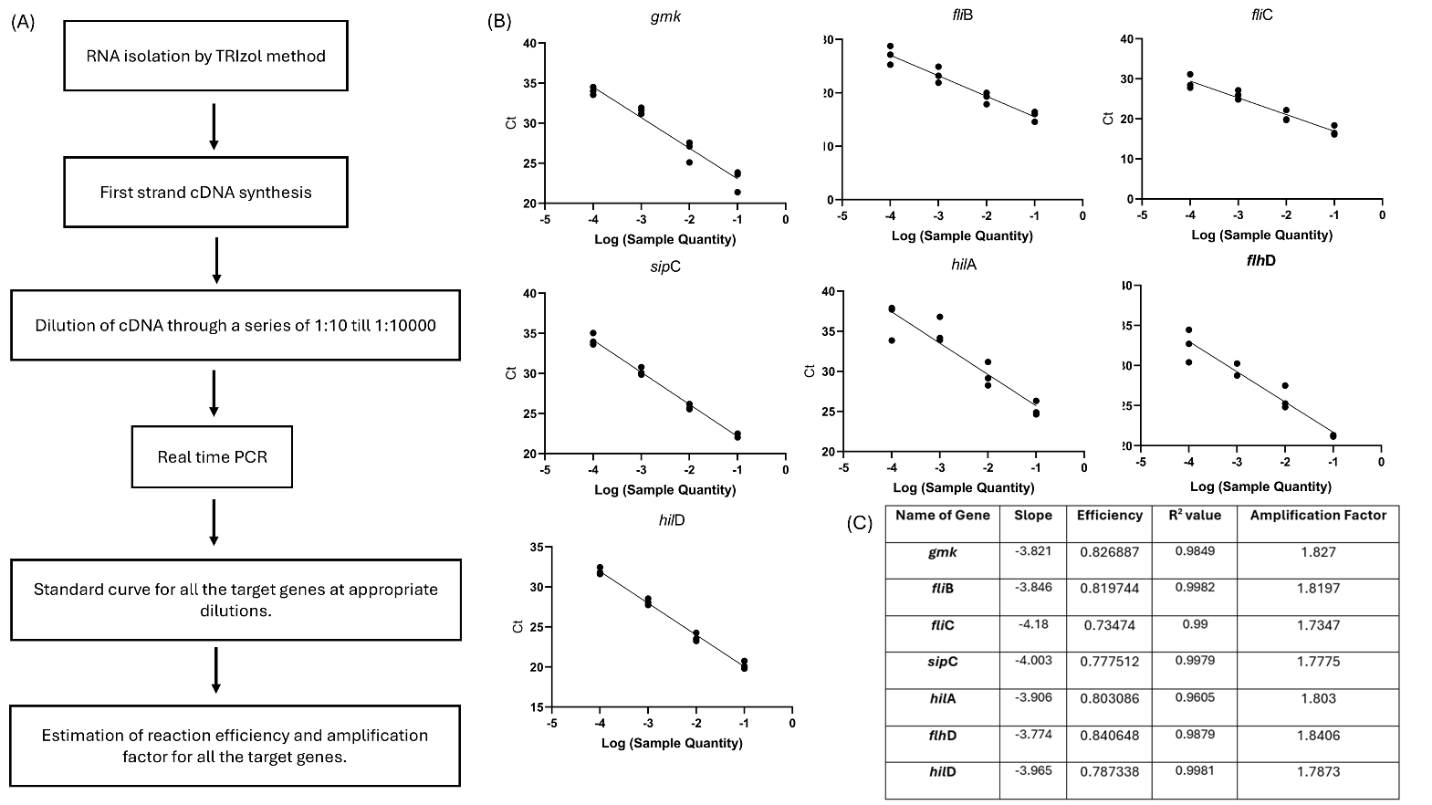


**Figure S4. qRT PCR primer efficiency was estimated for *gmk*, *fli*B, *fli*C, *sip*C, *hil*A, *flh*D, *hil*D.** Primer efficiency was estimated (A) using the protocol. (B) The quantitative real time PCR was run for all the target genes and (C) efficiency and amplification factor was calculated. Data are representative of 3 independent experiments. The slope was calculated using the linear regression line showing the relationship between the Ct value and cDNA concentration in the log scale.


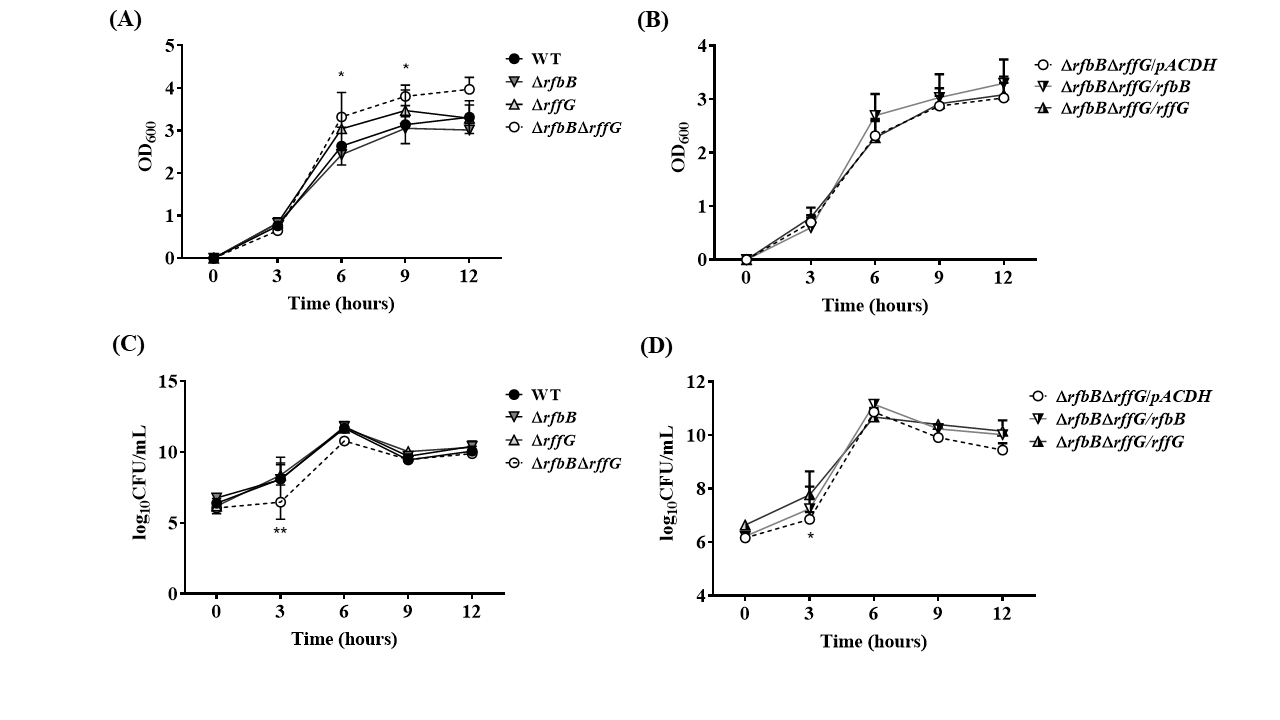


**Figure S5.** ***S*. Typhimurium Δ*rfbB*Δ*rffG*** **shows higher O.D. as compared to the WT and the single deletion mutants.** **(A)** *S*. Typhimurium WT, Δ*rfbB,* Δ*rffG*, and Δ*rfbB*Δ*rffG* were grown in LB broth for the indicated time points. **(B)** Growth of *S*. Typhimurium Δ*rfbB*Δ*rffG* with plasmid alone or either *rfbB* or *rffG* expressed *in trans*. Briefly, overnight grown cultures were normalized to O.D. 2 at 600 nm and 0.2% bacterial culture was inoculated in an Erlenmeyer flask containing 50 ml LB broth. The flasks were incubated at 37ºC under shaking conditions (160 rpm) for 12 hours. At indicated time intervals, 1 ml of the bacterial culture from each flask was aspirated and the O.D. was measured at 600 nm. **(C)** *S*. Typhimurium WT, Δ*rfbB*, Δ*rffG*, Δ*rfbB*Δ*rffG* and **(D)** complemented strains, Δ*rfbB*Δ*rffG*/pACDH, Δ*rfbB*Δ*rffG/rfbB*, Δ*rfbB*Δ*rffG/rffG* were grown in 50 ml LB broth at 37ºC. Culture aliquots were aspirated at indicated time points and appropriate dilutions were plated on LB agar plates. The plates were incubated at 37ºC, and the colonies obtained were enumerated. Data shown are representative of 3 independent experiments, plotted as mean ± SEM. Statistical analysis was performed using two-way ANOVA, where * *p* < 0.05 and ** *p* < 0.01.


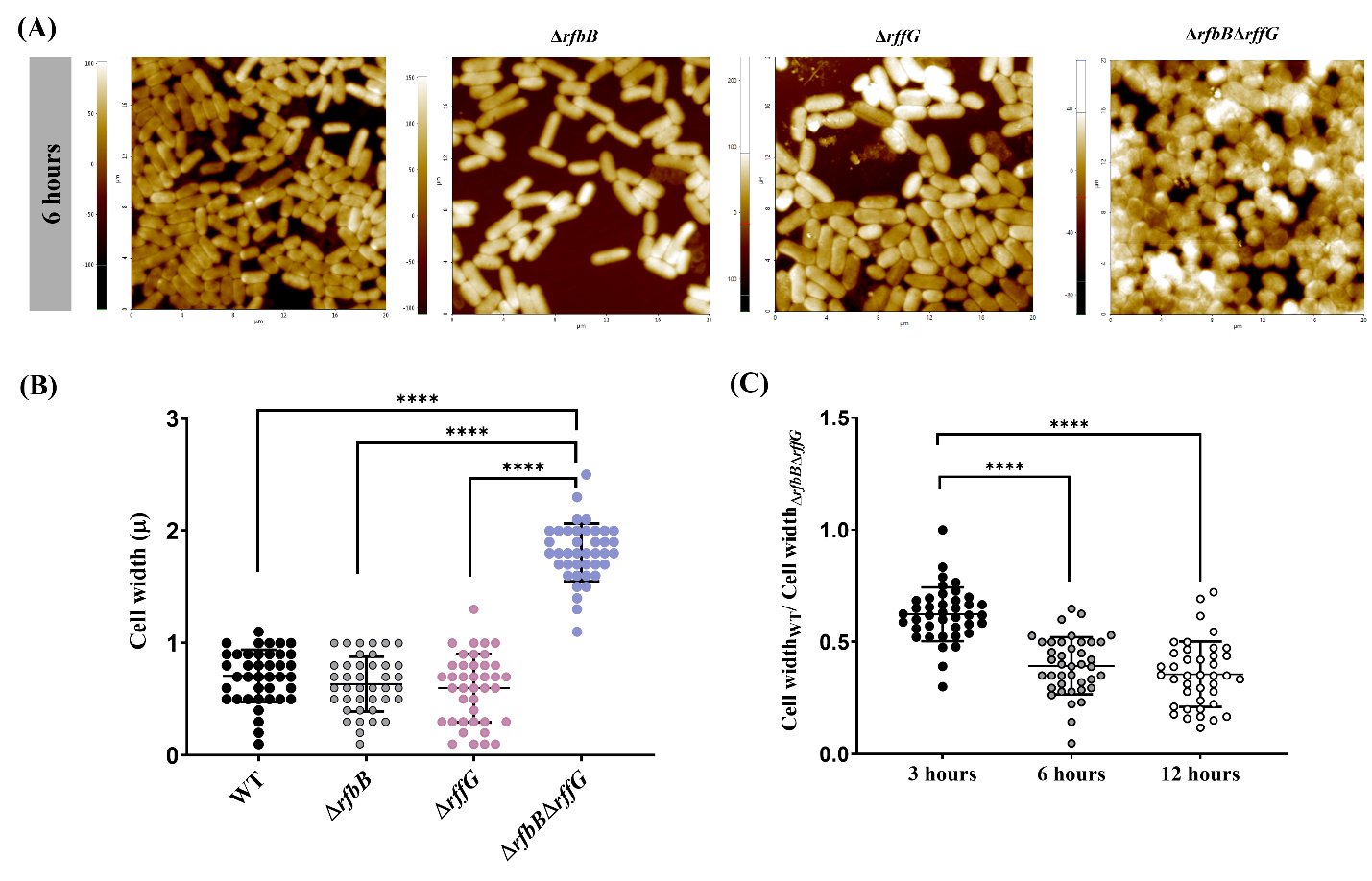


**Figure S6. *S*. Typhimurium Δ*rfbB*Δ*rffG*** **are more rounded in morphology than the WT and single deletion strains. (A)** WT, Δ*rfbB,* Δ*rffG* and Δ*rfbB*Δ*rffG* strains were grown for 6 hours and AFM images were acquired. **(B)** The cell width of at least 50 cells in each condition were measured and represented after 6 hours of growth in LB broth. **(C)** Ratio of cell width of WT: Δ*rfbB*Δ*rffG* strains estimated across time points. Data are representative of three independent experiments and is plotted as mean ± SEM. Statistical analysis was performed using two-way ANOVA, where **** *p* < 0.0001.


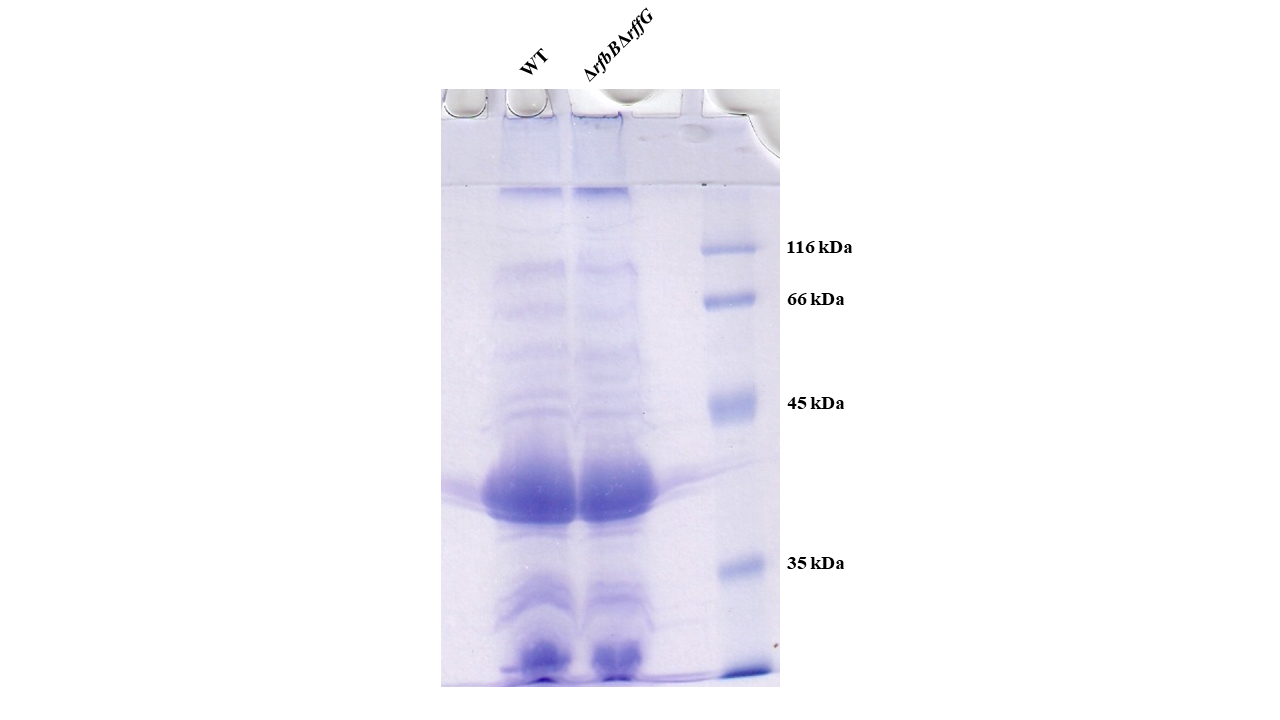


**Figure S7. Outer membrane protein (OMP) profile of *S*. Typhimurium WT and Δ*rfbB*Δ*rffG*.** *S*. Typhimurium WT and Δ*rfbB*Δ*rffG* were grown at 37ºC for 15 hours. The OMPs were isolated and resolved on a 12% SDS PAGE.


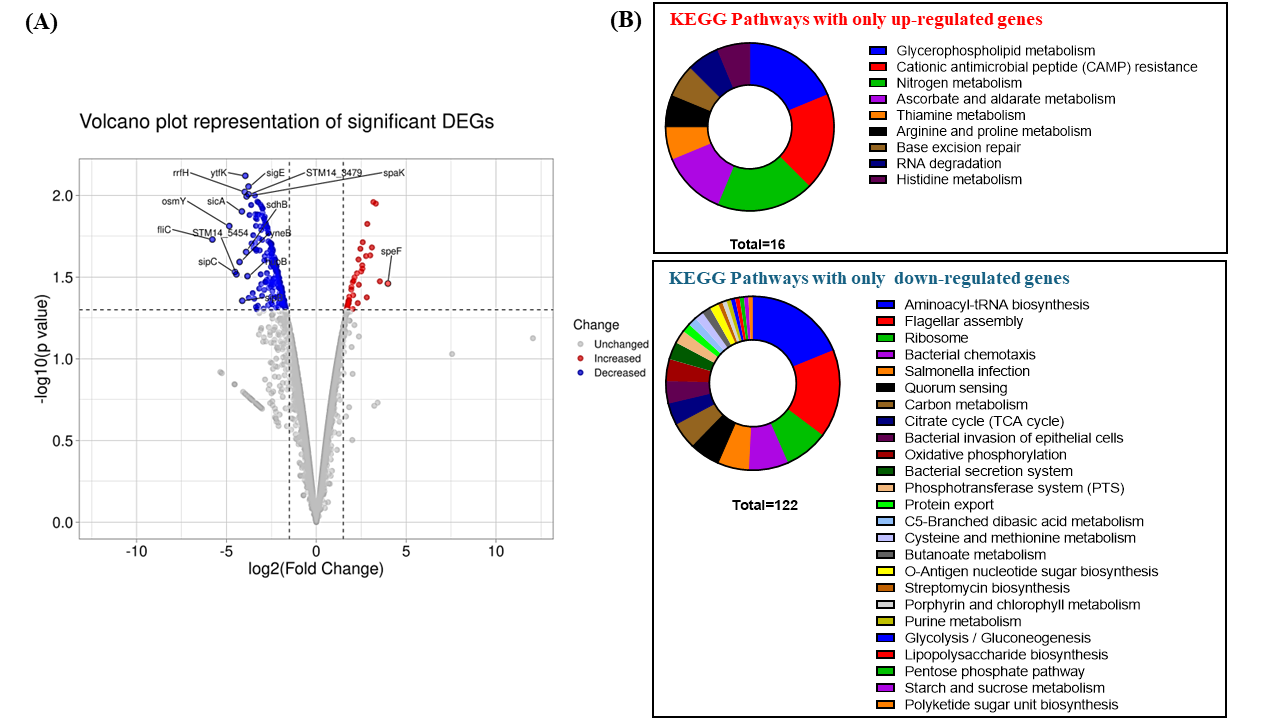


**Figure S8.** **RNAseq analysis depicts significantly more downregulated pathways in the *S.* Typhimurium Δ*rfbB*Δ*rffG*** **strain. (A)** Volcano plot representation of Differentially Expressed Genes (−1.5 ≤ log_2_Fold Change ≥ 1.5) in the Δ*rfbB*Δ*rffG* strain compared to the isogenic WT. **(B)** KEGG pathway enrichment of the significantly upregulated or downregulated genes in the Δ*rfbB*Δ*rffG* strain of *S*. Typhimurium.


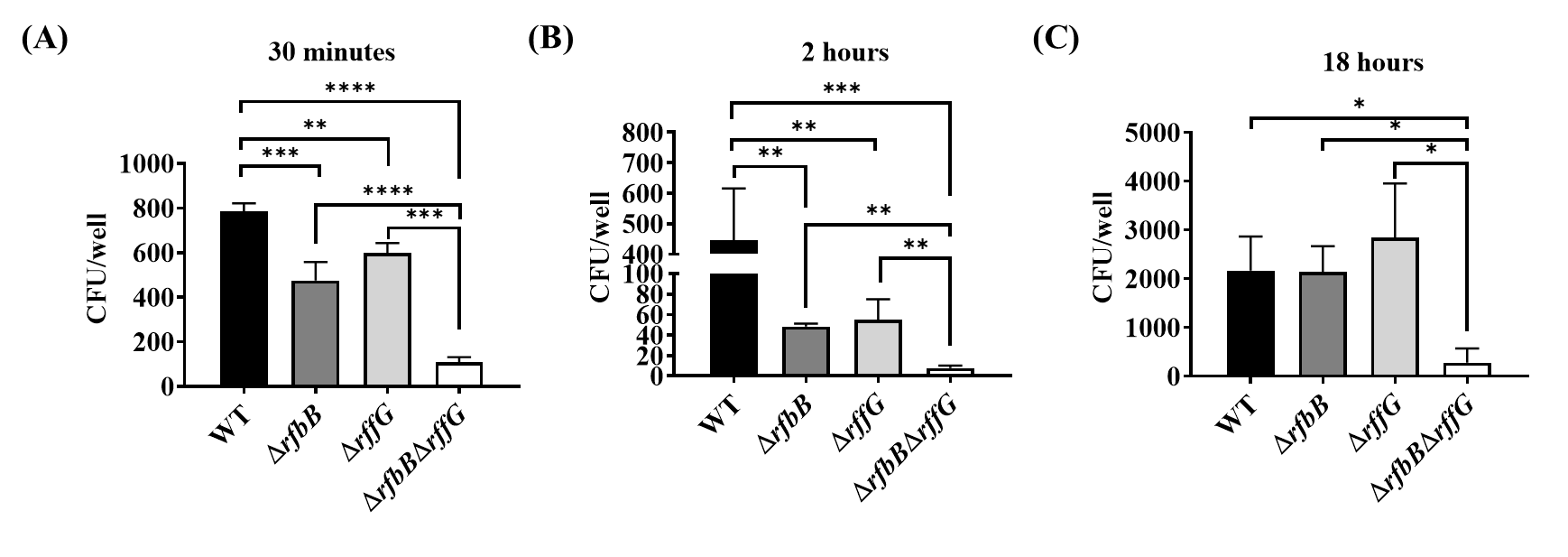


**Figure S9. *S.* Typhimurium Δ*rfbB,* Δ*rffG* and Δ*rfbB*Δ*rffG* are compromised with respect to adhesion and invasion in HeLa cells**. HeLa cells were infected at an MOI 1:10 and **(A)** adhesion, **(B)** invasion and **(C)** intracellular replication were studied at 30 minutes, 2 hours, and 18 hours post infection, respectively. Data are representative of 3 independent experiments expressed as mean ± SEM. Statistical analysis was performed using one-way ANOVA, where * *p* < 0.05; ** *p* < 0.01; *** *p* < 0.001 and **** *p* < 0.0001.

## Supplementary methods

**Multiple Sequence Alignment**

The protein sequences, RfbB (361aa; locus id: STM14_2591) and RffG (355aa; locus id: STM14_4720) from *S*. Typhimurium 14028s were obtained from the NCBI Protein records. These sequences were independently used as query sequences to perform protein BLAST (using the NCBI BLASTp suite) against the non-redundant (NR) sequence database. To identify close homologs, the search set was curated and limited to records from reference genomes of prokaryotic and eukaryotic representative organisms. BLAST was performed with the default algorithm parameters. The hits obtained from the above analysis were further filtered and only homologs with highest percentage identity in each reference genome were taken for further analysis. The FASTA sequences producing significant alignment with the input query sequence were downloaded and used as an input in the CLUSTAL Omega program (Sievers et al., 2011). CLUSTAL Omega is a Multiple Sequence Alignment (MSA) program that uses seeded guide trees and HMM profile-profile techniques to generate alignments between three or more sequences. For visualization and analysis of the alignment, JalView (Waterhouse et al., 2009) was used to highlight important features in the alignment, e.g., the conserved residues, and generating a consensus sequence.

**Library preparation, RNA sequencing, data analysis and KEGG pathway enrichment**

For each sample, 1 µg of total RNA was taken as input for RiboMinus Transcriptome Isolation Kit (Thermo Fisher Scientific) to remove ribosomal RNA. The ribosomal RNA depleted samples were used to generate a sequencing library using NEB NEXT RNA Ultra II library preparation kit for Illumina. Briefly, RNA was fragmented, and reverse transcribed to generate cDNA. Hairpin adapters were ligated to fragmented double-strand cDNA and USER enzyme was used to cleave the hairpin structure. Ampure beads were used to purify adapter-ligated fragments and the purified product was amplified using Illumina Multiplex Adapter primers to generate a sequencing library with barcodes for each sample. The library was quantitated using Qubit DNA High Sensitivity quantitation assay and library quality was checked on the Bioanalyzer 2100 using the Agilent 1500 DNA Kit. The QC passed libraries were diluted to 2 nM and pooled together. The pooled library was further diluted to sequence it on Illumina Hiseq as per the manufacturer’s recommendation. The Hiseq Control Software was used to setup 2x150 bp run on Hiseq and data was demultiplexed using bcl2fastq v2.1.9. The sequence data quality was checked using FastQC and MultiQC (Ewels et al., 2016) software. The data was checked for base call quality distribution, % bases above Q20, Q30, %GC, and sequencing adapter contamination. All samples passed the QC threshold (Q30 > 80%). Raw sequence reads were processed to remove the adapter sequences and low-quality bases using Trim Galore. Alignment and expression analysis: The QC passed reads were mapped onto indexed *S*. Typhimurium 14028s strain reference genome using HISAT2 (Kim et al., 2015, Kim et al., 2019) aligner. On average 97.53% of the reads aligned onto the reference genome. The PCR and optical duplicates were marked and removed using Picard tools (Broad Institute). Gene level expression values were obtained as read counts using featureCounts software (Liao et al., 2014). For differential expression analysis, DESeq2 (Love et al., 2014, Liu et al., 2021) package was used. Genes with < 5 reads in any one of the samples per condition were removed. The read counts were normalized (variance stabilized normalized counts) and differential expression analysis was performed. All the samples were compared to WT untreated condition independently. Genes with absolute log2 fold change ≥ 1.5 and p-value ≤ 0.05 (Wald’s test) were considered significant. The expression profile of the DEGs across the samples are presented as volcano plots using the VolcaNoseR web app (Goedhart and Luijsterburg, 2020). Gene set enrichment analysis was performed with the significant DEGs for pathway enrichment using the KEGG mapper tool (Kanehisa and Sato, 2020, Kanehisa et al., 2022).

**Outer membrane protein isolation, purification, and quantitation**

OMPs were isolated from the *S*. Typhimurium strains grown in LB (Ray et al., 2019). Briefly, cells were harvested in the late-log phase (12 hours) of growth and washed twice with 1X PBS. Approximately O.D. 4.0 (600 nm) cells were used for the extraction. OMP concentrations were determined by the Bradford’s assay, using BSA as standard. Equal volume of resuspended solution was loaded and analyzed by a 12.5% SDS-PAGE and visualized by staining with Coomassie Brilliant Blue (Sigma).

**Adhesion, invasion, and intracellular replication assay**

HeLa cells were cultured in Dulbecco's Modified Eagle Medium (DMEM) containing 4.5 g/L D-glucose, 4 mM L-glutamine and 1.5 g/L sodium bicarbonate (HiMedia, Mumbai, India) supplemented with 10% (v/v) FBS, 5% CO_2_ at 37°C. Approximately 24 hours before initiating the experiment the cells were seeded at the density of 1.5x10^4^ cells/well in a 96-well plate. Bacterial cells were grown overnight at 37°C. The absorbance of the overnight grown pre-inoculum was normalized to OD 2.0 at 600 nm and 50 μl of this pre-inoculum was inoculated in 50 ml of LB and grown at 37°C for 10 hours (Eriksson et al., 2003, Wu et al., 2014). For adhesion assay, both the inoculum and the 96-well plates were kept on ice for 15 minutes prior to infection. Subsequently, cells were infected at an MOI 1:10 and incubated on ice for 30 minutes. Cells were then washed thrice with sterile PBS, lysed using 100μl of 0.1% Triton X-100, and appropriate dilutions were plated on LB agar plates. For invasion and intracellular replication assays, HeLa cells were infected with an MOI 1:10 and 96-well plates were centrifuged at 2000 rpm of 2 minutes. Following incubation at 37°C for 50 minutes, the cells were washed twice with PBS and 100 μl of 100 μg/ml of gentamycin made in DMEM was added to kill the extracellular bacteria. To study invasion, cells were lysed with 0.1% Triton X-100 at 2 hours post infection, and appropriate dilutions were plated on LB agar. Following 2 hours, the gentamycin concentration was reduced to 25 μg/ml and cells were incubated at 37°C. At 18 hours post infection, gentamycin-containing medium was aspirated, and the cells washed twice with PBS followed by the addition of 0.1% Triton X-100 to the wells. Appropriate dilutions were plated on LB agar plates to determine the intracellular bacterial load.

## Supplementary references

Allam, U. S., Krishna, M. G., Lahiri, A., Joy, O. & Chakravortty, D. 2011. Salmonella enterica serovar Typhimurium lacking hfq gene confers protective immunity against murine typhoid. *PLoS One,* 6**,** e16667.

Bouhss A, Trunkfield AE, Bugg TD, Mengin-Lecreulx D. 2008. The biosynthesis of peptidoglycan lipid-linked intermediates. FEMS Microbiol Rev. 32, 208-33.

Canals, R., Hammarlof, D. L., Kroger, C., Owen, S. V., Fong, W. Y., Lacharme-Lora, L., Zhu, X., Wenner, N., Carden, S. E., Honeycutt, J., Monack, D. M., Kingsley, R. A., Brownridge, P., Chaudhuri, R. R., Rowe, W. P. M., Predeus, A. V., Hokamp, K., Gordon, M. A. & Hinton, J. C. D. 2019. Adding function to the genome of African Salmonella Typhimurium ST313 strain D23580. *PLoS Biol,* 17**,** e3000059.

Eriksson, S., Lucchini, S., Thompson, A., Rhen, M. & Hinton, J. C. D. 2003. Unravelling the biology of macrophage infection by gene expression profiling of intracellular Salmonella enterica. *Molecular Microbiology,* 47**,** 103-118.

Ewels, P., Magnusson, M., Lundin, S. & Käller, M. 2016. MultiQC: summarize analysis results for multiple tools and samples in a single report. *Bioinformatics,* 32**,** 3047-3048.

Goedhart, J. & Luijsterburg, M. S. 2020. VolcaNoseR is a web app for creating, exploring, labeling and sharing volcano plots. *Sci. Rep.,* 10**,** 20560.

Kanehisa, M. & Sato, Y. 2020. KEGG Mapper for inferring cellular functions from protein sequences. *Protein Sci.,* 29**,** 28-35.

Kanehisa, M., Sato, Y. & Kawashima, M. 2022. KEGG mapping tools for uncovering hidden features in biological data. *Protein Science,* 31**,** 47-53.

Kim, D., Langmead, B. & Salzberg, S. L. 2015. HISAT: a fast spliced aligner with low memory requirements. *Nat. Methods,* 12**,** 357-360.

Kim, D., Paggi, J. M., Park, C., Bennett, C. & Salzberg, S. L. 2019. Graph-based genome alignment and genotyping with HISAT2 and HISAT-genotype. *Nat. Biotechnol.,* 37**,** 907-915.

Kong, Q., J. Yang, Q. Liu, P. Alamuri, K. L. Roland and R. Curtiss. 2011. Effect of deletion of genes involved in lipopolysaccharide core and O-antigen synthesis on virulence and immunogenicity of Salmonella enterica serovar typhimurium. Infect Immun 79, 4227-4239.

Liao, Y., Smyth, G. K. & Shi, W. 2014. featureCounts: an efficient general purpose program for assigning sequence reads to genomic features. *Bioinformatics,* 30**,** 923-930.

Little K, Tipping Mj, Gibbs Ka. 2018. Swarmer Cell Development of the Bacterium Proteus mirabilis Requires the Conserved Enterobacterial Common Antigen Biosynthesis Gene rffG. J Bacteriol. 200, e00230-18.

Liu, S., Wang, Z., Zhu, R., Wang, F., Cheng, Y. & Liu, Y. 2021. Three differential expression analysis methods for RNA sequencing: Limma, EdgeR, DESeq2. *J. Vis. Exp.*

Love, M. I., Huber, W. & Anders, S. 2014. Moderated estimation of fold change and dispersion for RNA-seq data with DESeq2. *Genome Biol.,* 15**,** 550.

Marolda, C. L. and M. A. Valvano. 1995. Genetic analysis of the dTDP-rhamnose biosynthesis region of the Escherichia coli VW187 (O7:K1) rfb gene cluster: identification of functional homologs of rfbB and rfbA in the rff cluster and correct location of the rffE gene. J Bacteriol. 177, 5539-5546.

Parakkottil Chothi M, Duncan GA, Armirotti A, Abergel C, Gurnon JR, Van Etten JL, Bernardi C, Damonte G, Tonetti M. 2010. Identification of an L-rhamnose synthetic pathway in two nucleocytoplasmic large DNA viruses. J Virol. 84, 8829-38.

Ray, S., Da Costa, R., Das, M. & Nandi, D. 2019. Interplay of cold shock protein E with an uncharacterized protein, YciF, lowers porin expression and enhances bile resistance in <em>Salmonella</em> Typhimurium. *Journal of Biological Chemistry,* 294**,** 9084-9099.

Sievers, F., Wilm, A., Dineen, D., Gibson, T. J., Karplus, K., Li, W., Lopez, R., Mcwilliam, H., Remmert, M., Söding, J., Thompson, J. D. & Higgins, D. G. 2011. Fast, scalable generation of high-quality protein multiple sequence alignments using Clustal Omega. *Molecular Systems Biology,* 7**,** 539.

Spöring, I., Felgner, S., Preuße, M., Eckweiler, D., Rohde, M., Häussler, S., Weiss, S. & Erhardt, M. 2018. Regulation of Flagellum Biosynthesis in Response to Cell Envelope Stress in Salmonella enterica Serovar Typhimurium. *mBio,* 9.

Waterhouse, A. M., Procter, J. B., Martin, D. M. A., Clamp, M. & Barton, G. J. 2009. Jalview Version 2—a multiple sequence alignment editor and analysis workbench. *Bioinformatics,* 25**,** 1189-1191.

Wu, J., Pugh, R., Laughlin, R. C., Andrews-Polymenis, H., Mcclelland, M., Bäumler, A. J. & Adams, L. G. 2014. High-throughput assay to phenotype Salmonella enterica Typhimurium association, invasion, and replication in macrophages. *J. Vis. Exp.***,** e51759.
